# Systematic Moiety Variations of Ultrashort Peptides Produce Profound Effects on Self-Assembly, Nanostructure Formation, Hydrogelation, and Phase Transition

**DOI:** 10.1038/s41598-017-12694-9

**Published:** 2017-10-10

**Authors:** Kiat Hwa Chan, Bo Xue, Robert C. Robinson, Charlotte A. E. Hauser

**Affiliations:** 1 Institute of Bioengineering and Nanotechnology, Biopolis, A*STAR (Agency for Science, Technology and Research), Singapore, 138669 Singapore; 2 Division of Science, Yale-NUS College, 16 College Avenue West, Singapore, 138527 Singapore; 3 Institute of Molecular and Cell Biology, Biopolis, A*STAR (Agency for Science, Technology and Research), Singapore, 138673 Singapore; 4 NUS Synthetic Biology for Clinical and Technological Innovation, Centre for Life Sciences, National University of Singapore, 28 Medical Drive, Singapore, 117456 Singapore; 5 Department of Biochemistry, Yong Loo Lin School of Medicine, National University of Singapore, 8 Medical Drive, Singapore, 117597 Singapore; 6 Research Institute for Interdisciplinary Science, Okayama University, Okayama, 700-8530 Japan; 7 Laboratory for Nanomedicine, King Abdullah University of Science and Technology, Thuwal, 23955-6900 Saudi Arabia

## Abstract

Self-assembly of small biomolecules is a prevalent phenomenon that is increasingly being recognised to hold the key to building complex structures from simple monomeric units. Small peptides, in particular ultrashort peptides containing up to seven amino acids, for which our laboratory has found many biomedical applications, exhibit immense potential in this regard. For next-generation applications, more intricate control is required over the self-assembly processes. We seek to find out how subtle moiety variation of peptides can affect self-assembly and nanostructure formation. To this end, we have selected a library of 54 tripeptides, derived from systematic moiety variations from seven tripeptides. Our study reveals that subtle structural changes in the tripeptides can exert profound effects on self-assembly, nanostructure formation, hydrogelation, and even phase transition of peptide nanostructures. By comparing the X-ray crystal structures of two tripeptides, acetylated leucine-leucine-glutamic acid (Ac-LLE) and acetylated tyrosine-leucine-aspartic acid (Ac-YLD), we obtained valuable insights into the structural factors that can influence the formation of supramolecular peptide structures. We believe that our results have major implications on the understanding of the factors that affect peptide self-assembly. In addition, our findings can potentially assist current computational efforts to predict and design self-assembling peptide systems for diverse biomedical applications.

## Introduction

Self-assembly is a ubiquitous process in Nature that is responsible for the dynamic building of diverse biological structures, ranging from phospholipid-based cell membranes, and condensation of nucleic acid fibres, to the tubulin-based microtubules^1^. Biological self-assembly stems from the ability of the biomolecule to interact with one another via ‘recognition motifs’ unique to the biomolecule. The power of self-assembly to engineer complex biomolecular structures has been extensively exploited^2^. Two conceptual strategies were recently proposed, where self-assembly via specific inter-/intramolecular interactions and molecular shape complementarity points to the possibility to translate the information encoded in simple monomeric units via the ‘folding’ or ‘puzzle’ approaches into information-rich complex functional multimeric structures^3^. In the ‘puzzle’ approach, simple monomeric units are assembled into higher-order functional structures via the same recognition motif (e.g. a phospholipid bilayer). On the other hand, the ‘folding’ approach assembles a long sequence via similar interaction motifs into a functional unit (e.g. proteins). A supramolecular complex structure, like a ribosomal complex, represents the outcome, and potential, of harnessing both the ‘folding’ and ‘puzzle’ approaches^4^.

Information encoded in monomeric proteins and peptides can also be translated into macroscopic functions^5^. The ability of certain proteins and peptides to self-assemble and aggregate is well known and has been studied extensively^6,7^. Medically prominent examples of protein/peptide aggregation leading to diseases include human islet amyloid polypeptide of Type II diabetes mellitus^8,9^, prion proteins of Creutzfeldt-Jakob disease^10–12^, and amyloid peptide of Alzheimer’s disease^13,14^. In these instances, the accumulation of protein and peptide aggregates in the islet or brain cells have been implicated as the cause of cell death and consequently disease progression. Naturally, studies have been carried out to understand the mechanism of protein and peptide aggregation and aromatic amino acid residues have been proposed to initiate and propagate the formation of cytotoxic peptide aggregates^15,16^, although studies by Hauser *et al*. and Lakshmanan *et al*. challenge this notion^17,18^. Based on the understanding that aromatic residues govern the self-assembly behavior, efforts have also been devoted to design inhibitors that can potentially arrest the formation of toxic amyloid fibrils, thus delaying or even halting the onset of Alzheimer’s disease^19,20^.

However, the deleterious phenomenon of amyloid formation is only one aspect of peptide self-assembly. There are a variety of physiological situations where peptide self-assembly and aggregation are necessary phenomena, such as peptide hormone storage or microbial biofilm formation^21^. Due to the facile synthesis and functionalisation of peptides, they can be easily exploited in the design and fabrication of functional architectural mimics of desired extracellular matrices^22^, nanomaterials^23^, nanodevices and biosensors^21,24^. Through the ability of self-assembled peptide fibrils to entrap water, peptides have also been utilized in biomedical applications as hydrogels^25–27^. The sources of these peptides include natural proteins (e.g. elastin, collagen) as well as synthetic peptides^28^. Designer synthetic peptide sequences, such as RADA16, show how well-defined synthetic oligopeptides can be used to prepare hydrogels^29^. Unlike proteins derived from animal and plant sources, synthetic peptides prepared from natural amino acids can be easily purified with no concern about biological contamination. In addition, the composition of synthetic peptides is well defined and peptide properties can easily be tuned at the molecular level. As demonstrated elegantly by De Santis *et al*., alteration of peptide length and net charge can lead to changes in peptide self- assembled nanostructures that in turn elicit different biological responses^30^. Indeed, peptide hydrogels have been explored for many medical uses^31–36^, including, but not limited to, high throughput screening of anticancer drugs^37^, promotion of central nervous system regeneration^38^, hemostasis^39^, and angiogenesis via inclusion of VEGF-165 mimics in tissue engineering substrates^40^.

Recently, we have found that ultrashort peptides with a rationally designed amphiphilic sequence motif, which are similar in length to some self-assembling tripeptides^41–45^, have an innate tendency to also self-assemble into helical fibers and entrap water to form hydrogels^17,46^. Although numerous short peptides have been reported to form hydrogels, they comprise either aromatic residues (e.g. Phe) or aromatic functional groups (e.g Fmoc) that encourage self-assembly via *π*-*π* stacking interactions^47–54^. Our ultrashort peptides comprise only aliphatic residues and terminate in a polar amino acid, e.g. acetylated Leu-Ile-Val-Ala-Gly-Asp (Ac-LIVAGD) and acetylated Ile-Val-Asp (Ac-IVD). We have found that these ultrashort peptides are generally cytocompatible and the mechanical properties of the hydrogels can be easily tuned via minor sequence modifications, and changes in peptide and salt concentrations^46,55^. This makes our ultrashort peptides extremely suitable for biomedical applications, which include acceleration of wound healing^56,57^, delivery of anticancer cisplatin^58^ as well as antibacterial silver nanoparticles^59^.

The self-assembly of Ac-IVD has also been utilized to parameterize a computational algorithm for predicting ultrashort peptide aggregation^60^. In the process of validating the computational algorithm, the aggregation properties of six tripeptides were studied. In the process, we observed that single residue changes could exert major effects on the self-assembling properties on the peptides, as has been observed for other gelator systems^61–66^. As the single amino acid residue change leads to a drastic change in the side chain, we were curious if even smaller changes, i.e. on the molecular moiety scale in which the side chains are closely related to each other, can affect self-assembly of ultrashort peptides. In particular, as we had previously solved the crystal structure of Ac-YLD^60^, it could be possible to discern the effect of subtle structural changes on crystal packing. From our systematic moiety variations of seven ultrashort peptides (Ac-IVD, Ac-VIE, Ac-LVE, Ac-LLE, Ac-MYD, Ac-YLD, Ac-YYD), we discover that the self-assembly, nanostructure formation, hydrogelation, and phase transition of ultrashort peptides is intimately sensitive to small structural variations. Such a correlation can be attributed to the nanocrystalline origin of the fibril, which in turn is essential to the hydrogelation process.

## Results and Discussion

**Figure 1:**
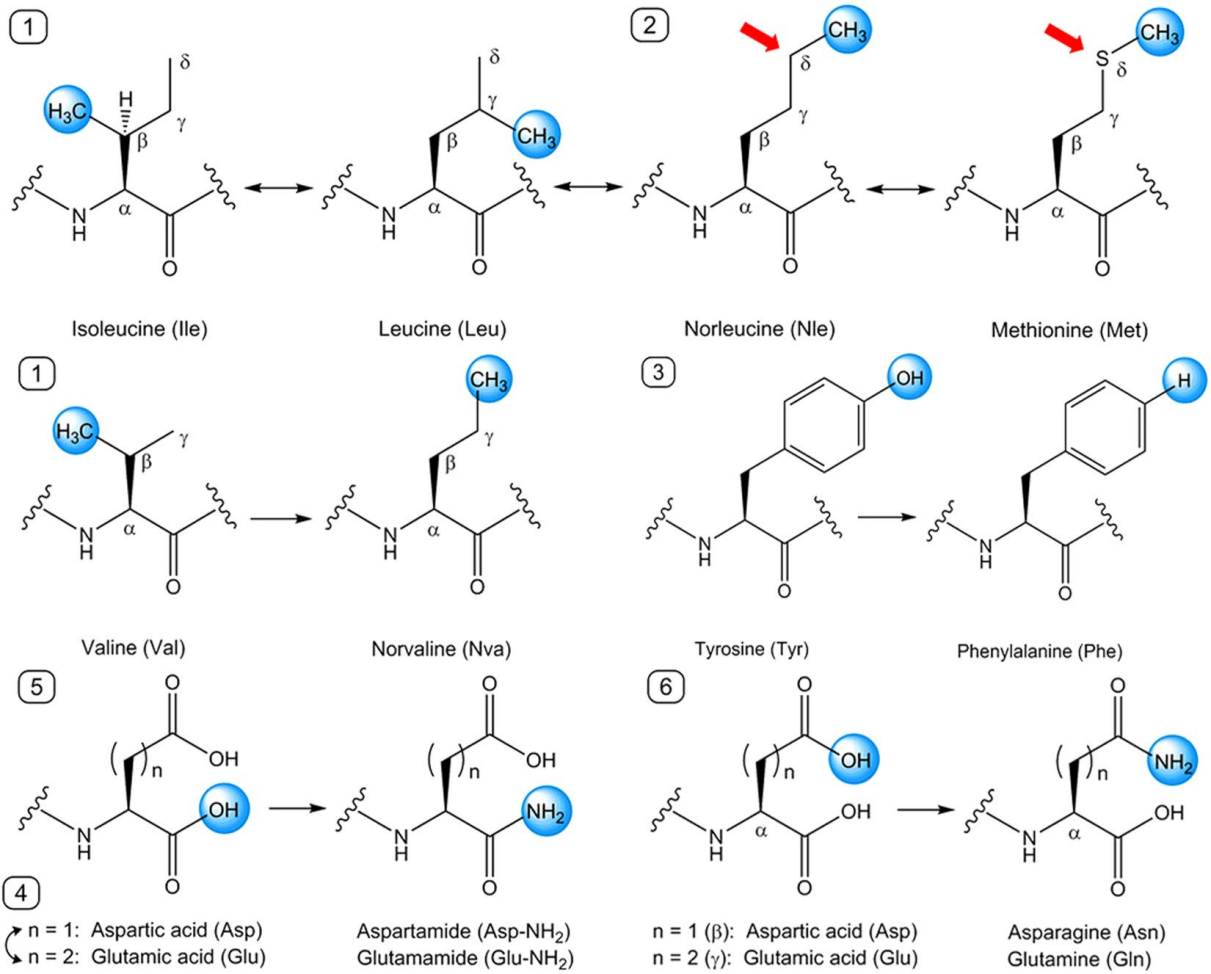
Illustration of the moiety variations carried out on a single amino acid residue at a time. 1: Me(*β* ↔ *γ* ↔ δ) shifts, 2) (CH_2_ ↔ S) interconversion, 3) para-Ph(OH→H) conversion, 4) n(1 ↔ 2) interconversion, 5) *β*/*γ*(CO_2_H → CONH_2_) conversion, 6) *α*(CO_2_H → CONH_2_) conversion.

Previously, in the process of verifying the capability of a computational algorithm to predict peptide aggregation, we investigated the self-assembly properties of six tripeptides (Ac-VIE, Ac-LVE, Ac-LLE, Ac-MYD, Ac-YLD, and Ac-YYD) alongside Ac-IVD^60^. These seven tripeptides, which are well characterised, can be categorised into two groups according to the nanostructures that they form after self-assembly: crystalline (Ac-LLE, Ac-LVE, Ac-YLD) and fibrillar (Ac-IVD, Ac-VIE, Ac-MYD, Ac-YYD). Previously, Ac-LVE was categorised as being fibrillar;^60^ on re-analysis, Ac-LVE is more accurately categorised as being crystalline here. Based on this categorisation, we explored and compared the effects of systematic single moiety variations on self-assembly of these seven parent tripeptides. The variations we considered are: 1) chain-isomeric methyl shifts on leucine and valine, 2) isosteric interconversion of methylene and sulfur (Nle ↔ S), 3) dehydroxylation of tyrosine, 4) homologous interconversion of Asp and Glu, 5) amidation of the C-terminus, and 6) amidation of the carboxyl side chains. Variations (1–3) affect hydrophobic interactions by residues in positions 1 and 2, i.e. P1 and P2; variations (4–6) affect hydrogen bonding by P3 residues. These moiety variations on the seven parent tripeptides are illustrated in Fig. 1 and generate the panel of tripeptides shown in Table 1. The profound effects of the single moiety variations on a fibrillar parent tripeptide (Ac-IVD) and a crystalline parent tripeptide (Ac-LLE) are described in detail as examples.

**Table 1:**
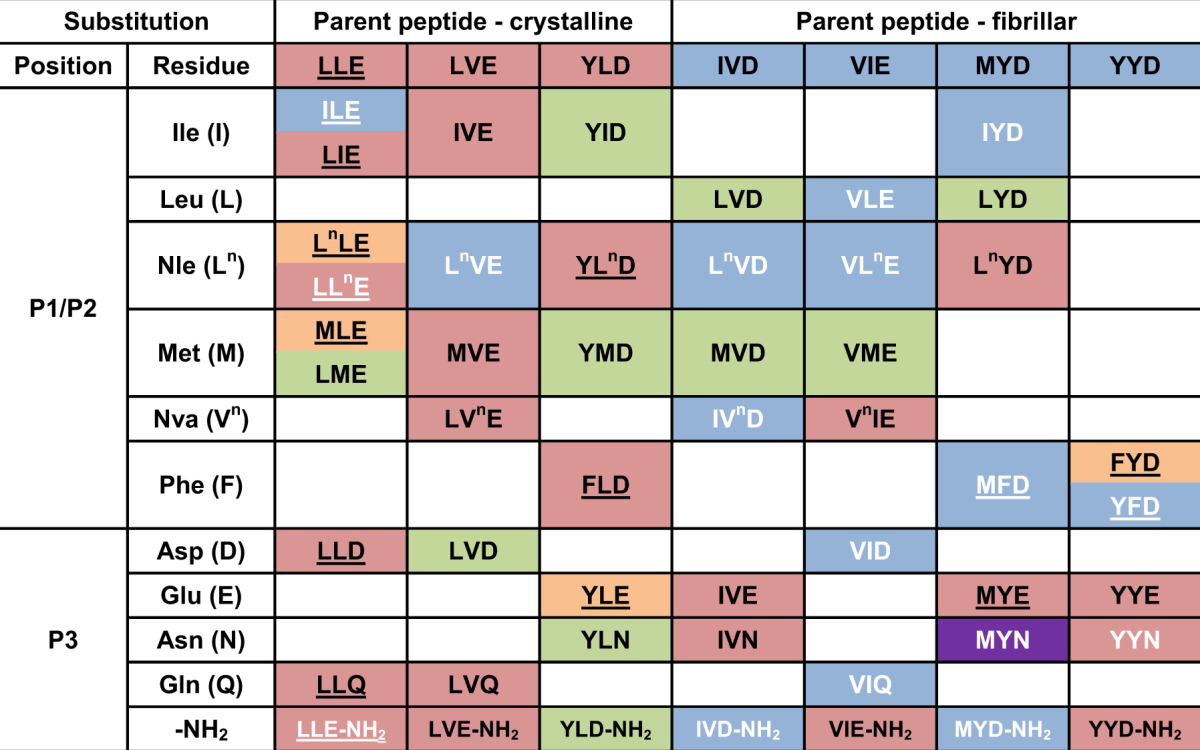
Ensemble of tripeptides derived from single moiety variations from seven parent peptides. All peptides are acetylated at the N-terminus. The colors denote the nanostructures adopted by the tripeptides upon self-assembly after two weeks: amorphous (green), bead (cream), crystalline (red), and fibrillar (blue). Purple indicates that Ac-MYN, depending on the duration of sonication, is able to adopt either crystalline or fibrillar nanostructure. The tripeptides that are in white form hydrogels. Underlined tripeptides indicate microbead formation.﻿

**Figure 2:**
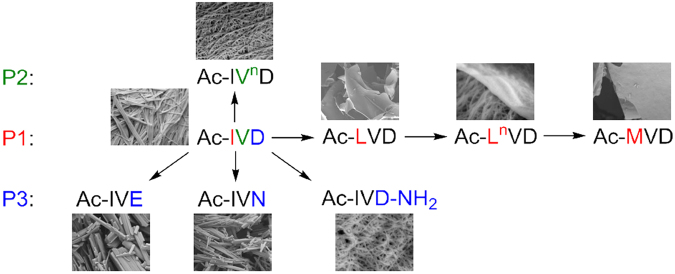
Depiction of the myriad changes in nanostructures that occurred when Ac-IVD was subjected to various moiety variation.

Ac-IVD self-assembles into fibrils that entrap water to form a hydrogel at 10–15 mg/mL^46^. P1(Ile → Leu) led to a total loss of self-assembly in Ac-LVD, as evident from the lack of fibrillisation at 5, 20, or 40 mg/mL. Yet, P1(Leu → Nle) restored self-assembly and hydrogelation in Ac-L^*n*^VD. Interestingly, small crystals of Ac-L^*n*^VD (at 20 mg/mL) were observed to form before hydrogelation set in. Such an observation has been made for dipeptide hydrogelators^67^. Finally, P1(Nle → Met) once again led to the loss of self-assembly in Ac-MVD, with no fibrils observed at even 40 mg/mL. P2(Val → Nva) also attenuated the entrapping of water in Ac-IV^*n*^D as hydrogelation concentration > 30 mg/mL (relative to Ac-IVD). However, self-assembly in Ac-IV^*n*^D was apparently unaffected, as evident from the fibrils observed in the hydrogel of Ac-IV^*n*^D. This is a remarkable series of changes, given that the changes are only due to chain-isomeric methyl shifts and isosteric (CH_2_ → S) conversion. P3(Asp → Glu) not only abolished gelation in Ac-IVE, but it also affected nanostructure formation – instead of the fibrils observed for Ac-IVD, Ac-L^*n*^VD, and Ac-IV^*n*^D, nanocrystallites were observed for Ac-IVE. Amidation of the C-terminus, i.e. P3(Asp → Asp-NH_2_), potentiated the entrapping of water in Ac-IVD-NH_2_ to gel at a lower hydrogelation concentration (*< *10 mg/mL). Yet, amidation of the carboxyl sidechain, i.e. P3(Asp → Asn), led instead to a loss of gelation in Ac-IVN. Moreover, the nanostructure formed by Ac-IVN (crystalline) is also distinctly different from that of Ac-IVD-NH_2_ (fibrillar), despite both peptides being positional isomers. These myriad changes are represented in Fig. 2.

**Figure 3:**
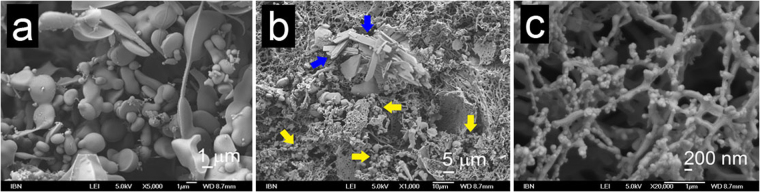
Scanning electron micrographs of Ac-ILE. (**a**) At 5 mg/mL, Ac-ILE dissolved completely and microbeads were formed; magnification 5000×. (**b**) At 20 mg/mL, Ac-ILE formed a hydrogel, with microbeads (yellow arrow), crystallites (blue arrow), and fibrils (dispersed throughout) being observed; magnification 1000×. (**c**) At 20 mg/mL, fusion of the beads into fibrils was observed; magnification 20000×. This suggests that the beads are precursors to fibril formation.

**Figure 4:**
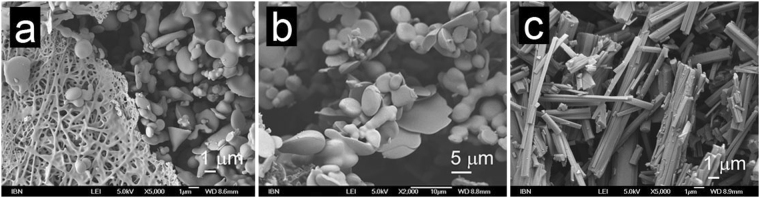
Scanning electron micrographs of Ac-LLE-NH_2_: (**a**) Hydrogel at 25 mg/mL illustrates the biphasic equilibrium between microbeads and fibrils; magnification 5000×. (**b**) Supernatant at 40 mg/mL; magnification 2000×. (**c**) precipitate at 40 mg/mL; magnification 5000×. B and C show that the microbeads can also co-exist with the crystalline phase.

Ac-LLE provides an excellent opportunity to compare and contrast the effect of the same conversion on positions P1 and P2. P1(Leu → Ile) imbued gelation in Ac-ILE whereas P2(Leu → Ile) did not have such an effect on Ac-LIE. While both the solution (Ac-ILE, 5 mg/mL) and supernatant (Ac-LIE, 5 mg/mL) exhibited bead microstructures, the hydrogel of Ac-ILE exhibited fibrillar nanostructures whereas the precipitate of Ac-LIE exhibited crystalline nanostructures. However, small crystals of Ac-ILE could also be observed to form, before hydrogelation set in. Interestingly, bead, crystalline and fibrillar structures were observed simultaneously in the hydrogel of Ac-ILE (20 mg/mL), suggesting that these three phases can co-exist together. In addition, the fusion of microbeads into fibrils was observed in the hydrogel of Ac-ILE (40 mg/mL; Fig. 3c). Similar fusions of Ac-A_6_D/Ac-V_6_D into nanotubes^68^ and of Boc-FF/FF nanobeads into “biomolecular necklaces”^69^ have been reported. Thus, our observations suggest that even aliphatic peptide-based nanobeads can serve as precursors to fibril formation. P1(Leu → Nle) had minimal effect on the properties of Ac-L^*n*^LE. Akin to Ac-LLE, Ac-L^*n*^LE was fully soluble (up to 40 mg/mL) and exhibited bead microstructures, although no crystallisation was observed. P2(Leu → Nle) accelerated the aggregation process so that Ac-LL^*n*^E started aggregating at 10 mg/mL. Microbeads were also observed at 5 mg/mL and in the supernatant at 20 mg/mL, along with crystallites in the aggregates (20 and 40 mg/mL). These observations indicate that the beads are the precursors to the crystallites. P1(Nle → Met) still led to self-assembly of Ac-MLE into bead microstructures, but with higher tendency to fuse into larger structures. This is also reflected in aggregation of these nanobeads (at 40 mg/mL) into crystalline aggregates after one month. However, P2(Nle → Met) completely abolished self-assembly, so that no discernible nanostructure was observed in Ac-LME even at 40 mg/mL. Evidently from these three sets of comparisons, the same variation can exert different effects on self-assembly and nanostructure formation at different positions of the peptide. Such dependence of self-assembly on the positional preference of certain residues in some tripeptides has also been reported by Frederix *et al*.^70^. As in Ac-LL^*n*^E, P3(Glu → Asp) accelerated the aggregation in Ac-LLD so that precipitation was observed starting at 20 mg/mL. However, unlike the gelatinous precipitate of Ac-LL^*n*^E, the precipitate of Ac-LLD was dense. Interestingly, the supernatant and precipitate of Ac-LLD exhibited also microbeads and nanocrystallites, respectively. While P3(Glu → Gln) accelerated slightly the self-assembly in Ac-LLQ, P3(Glu → Glu-NH_2_) led to gelation in Ac-LLE-NH_2_ at 25 mg/mL. However at higher concentrations of Ac-LLE-NH_2_ (30–40 mg/mL), small clumps of hydrogel were observed to form that quickly turned into an opaque precipitate. Analysis of the solutions (5 and 20 mg/mL), hydrogel (25 mg/mL), and precipitate (40 mg/mL) revealed three different morphologies: microbeads, nanofibrils, and nanocrystallites respectively (Fig. 4). Strikingly, both microbeads and nanofibrils were observed in the hydrogel (25 mg/mL) of Ac-LLE-NH_2_, directly illustrating their co-existence with each other (Fig. 4a).

**Figure 5:**
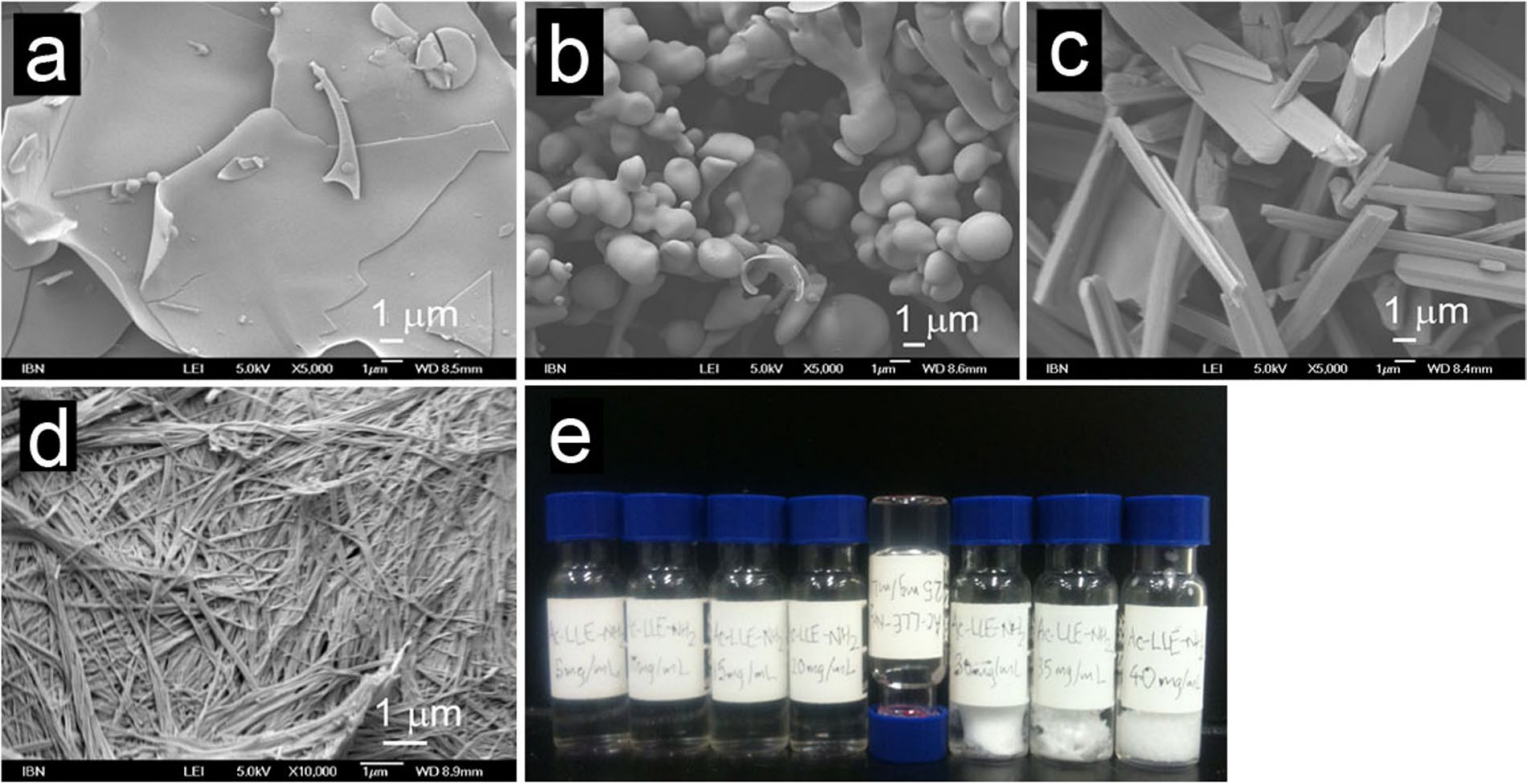
Scanning electron micrographs illustrating the four general morphologies observed with the acetylated tripeptides: (**a**) Amorphous structure (Ac-LVE, supernatant of 20 mg/mL; 5000×). (**b**) Bead microstructure (Ac-LLE, solution of 20 mg/mL; 5000×). (**c**) Crystalline nanostructure (Ac-LVE, precipitate of 20 mg/mL; 5000×). (**d**) Fibrillar nanostructure (Ac-IVD, hydrogel at 20 mg/mL; 10000×). (**e**) Illustration of a typical set-up to assess the self-assembly and aggregation of peptides. A series of peptide concentrations (Ac-LLE-NH_2_ here) from 5–40 mg/mL, in steps of 5 mg/mL were prepared. This series also illustrates the four states generally observed in this study: solution (5–20 mg/mL), hydrogel (25 mg/mL; upturned vial), supernatant and precipitate (30–40 mg/mL).

In general, the four basic morphologies exhibited by Ac-IVD/Ac-LLE and their variants are also observed with the other tripeptide variants generated via systematic single moiety variations: 1) amorphicity, 2) bead microstructures, 3) crystalline nanostructures, 4) fibrillar nanostructures (Fig. 5). This series of morphologies is very similar to those reported for the self-assembly of Boc-FF under various solvent conditions^71^. Amorphous structures are essentially featureless and do not exhibit any discernible nanostructure (Fig. 5a). Bead microstructures are rounded structures (microbeads) that can range from 400–2000 nm in width (Fig. 5b). Crystalline nanostructures (nanocrystallites) exhibit sharp edges akin to single crystals (Fig. 5c). Fibrillar nanostructures are long and thin fibrils of *∼*30 nm thick that make up the network within a hydrogel (Fig. 5d). The self-assembly/aggregation of the tripeptides was monitored across 5–40 mg/mL in pure water, in steps of 5 mg/mL (Fig. 5e). The remaining series of tripeptide preparations are presented in the Supporting Information. From our experience, this concentration range is sufficient to illustrate changes in aggregation of the tripeptides. The nanostructures formed by the tripeptides at 5, 20, and 40 mg/mL (low, medium, and high concentrations respectively) were then examined by field emission scanning electron microscopy (FESEM).

Our results clearly illustrate the profound impact that seemingly small moiety variations in a tripeptide structure can exert on self-assembly and nanostructure formation. The methyl shifts do not change the “hydrophobic content” of the peptide, but they can affect intermolecular peptide packing. As noted before, there is a difference in packing between Leu and Nle, in which Leu can pack more intimately than Nle^72^. As our crystal structure illustrates, the isobutyl side chains of Ile can interdigitate with each other to maximize hydrophobic interaction, a packing conformation inaccessible to Nle. Although Val possesses an isopropyl group that could potentially interdigitate, this is made unfavorable by the close proximity of the isopropyl group to the peptide backbone. The difference in packing of Leu and Val can be gleaned by comparing the self-assembly of Ac-LLE against Ac-LVE and Ac-VLE (Table 1). The packing in Ac-LVE was slightly affected compared to Ac-LLE so that self-assembly of Ac-LVE was much faster, but also led to smaller crystallites. In contrast, the packing of Ac-VLE is drastically affected so that while crystallites formed at 20 mg/mL (Figs S5-2), the crystallites transitioned into fibrils at higher concentrations. This is another example of the seemingly greater impact of P1 substitutions compared to P2 substitutions. The more efficient packing of Leu is amply illustrated by the two-dimensional self-assembly of tripeptides LLL and VVV on graphite^73^. Such differences in aliphatic chains are also well known to affect polypeptide helix formation^74^ and coiled-coil/leucine zipper formation^75^.

Even though Nle and Met have essentially the same chain lengths, as CH_2_ (Nle) and S (Met) are isosteric^76^, they exert drastically different effects on self-assembly and nanostructure formation. Based on lipid partitioning experiments, Nle- containing peptides have been found to be more hydrophobic than Met-analogs^77^. This could account for the general high solubility of Met-substituted tripeptides, with the exception of the Ac-MYD class. That a single substitution of CH_2_ with S, among numerous interactions possible in the rest of the tripeptide, abolishes self-assembly thus lends support to the criticality of C-H van der Waals interaction afforded by methylene (of Nle) toward self-assembly. Such interactions could account for the difference between A31-35/A25-35 peptides and their Nle analogs in affecting apoptotic cell death of PC12 cells^78^. As mentioned, the Ac-MYD class is exception to this rule – many analogs in the Ac-MYD class can readily self-assemble. This could be due to the Met-*π* interaction^79^, i.e. between the sulfur atom in Met and the aromatic ring in Tyr/Phe, which reduces the interaction of the sulfur atom with water and locks the tripeptide in position for ordered packing. The order of the residues is evidently important given that Ac-YMD does not self-assemble while Ac-MYD does.

The C-terminal carboxyl and the carboxyl side chain of Asp/Glu mediate critical hydrogen bond interactions, as illustrated by the crystal structures of Ac-LLE and Ac-YLD (previously-determined^60^). Amidation of carboxyl eliminates the hydrogen bond acceptor oxygen, and increases the number of hydrogen bond donors by one or two, depending on whether the carboxyl is protonated. It also limits the bond rotation of the hydrogen bond donors (amide protons), so some ordering in the peptide-peptide and peptide-water hydrogen bonds can be expected. Indeed, this may account for the faster gelation of C-terminus amidated Fmoc-Phe derivatives relative to carboxyl analogs^63^. An ordering of hydrogen bonding may thus be expected to favor crystalline structures. However, amidation in this study resulted in the crystallisation of only Ac-VIE-NH_2_ and Ac-YYD-NH_2_, while abolishing crystallisation of Ac-YLD-NH_2_. The crystal structures of Ac-LLE and Ac-YLD seem to suggest that amidation would introduce little steric hindrance to the packing. As such, the influence of amidation on the kinetics of self-assembly might be interpreted by the modified interactions between the peptides and the solvent water molecules surrounding them, a correlation we have made before^55^.

### Gaining Insights Into Peptide Self-Assembly via X-ray crystal structure of Ac-LLE

**Figure 6:**
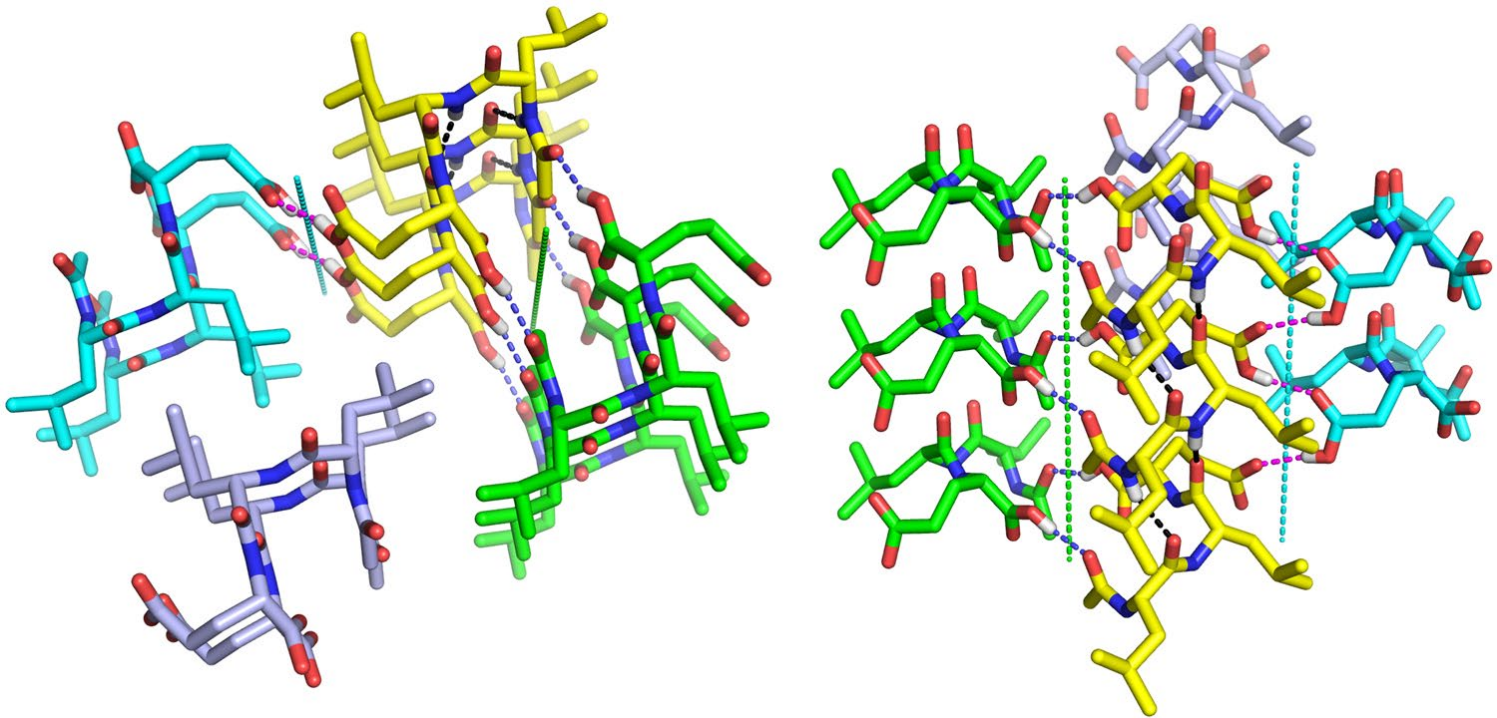
X-ray crystal structure of Ac-LLE. Four blocks of Ac-LLE are shown and colored differently. (Left) hydrophobic interaction by the intercalating side chains of Leu1 and Leu2. (Right) hydrogen bond network. Intra- and inter-block hydrogen bonds are colored black, blue and magenta, respectively. The 2-fold axis relating the yellow and the green blocks are in green, and the 2-fold screw axis relating the yellow and the cyan blocks are in cyan.

Previously, Ac-LLE was observed to be soluble in water up to 40 mg/mL, forming nanobeads in solution^60^. Recently, we observed that when Ac-LLE (30–40 mg/mL) was allowed to stand in a sealed vial for an extended period of time, long and thin needles slowly formed. Thus, an X-ray crystallographic study of Ac-LLE was carried out (Fig. 6 and Supplementary Table S1). The solved X-ray structure shows that the Ac-LLE crystal belongs to space group C2. The peptide backbone of Ac-LLE is significantly bent into a U-shape, which cause the peptides to stack on top of each other to form blocks. The blocks are laterally related by parallel 2-fold (screw) axes in the crystal. As a result, the two isopropyl side chains of Ac-LLE are able to intercalate with each other via hydrophobic interactions. Such an antiparallel arrangement of Ac-LLE supports the self-assembly configuration of ultrashort peptides previously proposed by Hauser *et al*.^17^. In addition to the hydrophobic interactions, there are prominent hydrogen bond networks in the crystal. Within the same block, the hydrogen bonds between the amide proton and amide carbonyl (2.03Å and 2.40Å, black) help to stitch together the U-shaped Ac-LLE. In-between the blocks, Ac-LLE peptides interact with each other via hydrogen bonds between the C-terminus carboxyl proton and acetyl carbonyl (1.84Å, blue), which are related by the 2-fold axis (green axis), as well as the carboxyl side chains of Glu (1.84Å, magenta), which are related by the 2-fold screw axis (cyan axis). The latter is in a zig-zag form, paired between the hydroxyl group of the protonated-carboxyl side chain (donor) and the adjacent carboxyl carbonyl (acceptor) that is related by the 2-fold screw axis. From the X-ray crystal structure of Ac-LLE, we can attempt to interpret the changes observed when we make single moiety variations on Ac-LLE.

The crystal structure of Ac-LLE, which was derived from crystals slowly grown in purely water, provides a useful guide to understanding how molecular moiety variations can influence self-assembly and nanostructure formation of the aliphatic tripeptides in water. The hydrophobic interaction arises from the tight packing of the isopropyl side chains of adjacent Ac-LLE molecules. This “hydrophobic space” is fairly tolerant to the single atomic variations at P2, given that Ac-LIE and Ac-LL^*n*^E still self-assemble into crystallites (Supplementary Fig. S1). P1, conversely, seems to have more impact on the self-assembly of Ac-LLE; while substitution by Ile enables Ac-ILE to gel, substitution by Nle greatly reduces self-assembly of Ac-L^*n*^LE. Substitution by Met at either P1 or P2 is unfavorable for self-assembly, which may be attributed to the increased surface entropy of Met, due to the greater number of rotatable bonds in the side chain, when compared with Leu and a decrease in hydrophobicity^80^. It is interesting to note that none of the modifications at P3 affect the crystallizability of the derived tripeptides, given that both the side chain and C-terminal carboxyls of Glu3 are critically involved in the inter-block hydrogen bonding (Fig. 6). Based on the crystal structure, the two amidations (Ac-LLQ and Ac-LLE-NH_2_) seem to be compatible with the crystal packing and the hydrogen-bonding pattern. To accommodate the shortening of side chain length in Ac-LLD, a shift in the position of the screw axis, with a concurrent change in the side chain conformation of Leu1 and Leu2, would probably be needed, resulting in a different crystal packing.

The previously determined crystal structure of Ac-YLD^60^ is valuable in explaining the observed phenomena for the series derived from Ac-YLD. The three modifications at P3 clearly violate the crystal packing requirements of Ac-YLD. The side chain of Asp3 is critical in forming the hydrogen bond network involving the carbonyl of the N-terminal acetyl and the water molecule that it coordinates, in which the protonated carboxyl acts as both hydrogen bond donor and acceptor. Changing Asp3 to Glu (Ac-YLE) or Asn (Ac-YLN) would lead to complete loss of this network. The protonated C-terminal carboxyl of Ac-YLD hydrogen bonds strongly with the carbonyl of Tyr1, with a bonding distance of 1.76 Å ; amidation of the C-terminus (Ac-YLD-NH_2_) turns the carboxyl into an amide, which would be less compatible with the surrounding environment in the crystal packing; even if it could still form a hydrogen bond to the carbonyl of Tyr1 via N-H:O, the bonding would be weaker than the original O-H:O. The hydrogen bond between the hydroxyl of Tyr1 and the carbonyl of the N-terminal acetyl is less critical, as it does not affect the above-mentioned hydrogen bond network. Consequently, loss of this hydrogen bond in the P1 (Tyr1 to Phe) substitution is well tolerated. For the three substitutions at P2, Nle is well tolerated (Ac-YL^*n*^D), which is likely due to its similar hydrophobicity to Leu2, as well as its high flexibility that permits a conformation compatible with the crystal packing. Despite being an isosteric substitution of Nle, Met, however, is not tolerated (Ac-YMD). Again, this may be the consequence of reduced hydrophobicity after substitution. Lastly, it was surprising to see that Ile is not tolerated by the crystal packing. This loss of self-assembly probably originates from the subtle difference in conformational flexibility between Ile and Leu^81^.

The (Tyr → Phe) substitutions manifest the effects of the hydroxyl group on the aromatic ring of the aromatic tripeptides in directing crystallisation. This conversion did not affect hydrogel formation in Ac-MFD (relative to Ac-MYD), but the speed of self-assembly was slower. For Ac-YLD and Ac-FLD, while both formed crystals, Ac-FLD had a much faster kinetics in nucleation, resulting in many nanocrystals instead of the fewer but larger microcrystals of Ac-YLD. Another case of altered kinetics was observed with P1(Tyr → Phe) of Ac-YYD: Ac-FYD was not observed to self-assemble, let alone fibrillise. P2(Tyr → Phe) of Ac-YYD provides an example of tuning into fibrillisation: Ac-YFD could form a transparent hydrogel at 15 mg/mL, whereas its parent Ac-YYD exhibited minimal gelation. This stands in stark contrast to the observations of Frederix *et al*., in which their unacetylated group of KFF, KYF, KYY (KFY was not reported) peptides all formed hydrogels^70^; in these cases, the unprotected N-termini could assist in peptide fibril solubility and consequently gelation. Thus, it appears that for Ac-YFD, the effects of the aromatic hydroxyl on the solubility and/or hydrogen bonding capability of the peptide are tuned ideally to permit nucleation and eventual fibrillisation. This finding highlights the importance of balancing the forces for/against self-assembly.

Ac-L^*n*^VD, Ac-VLE, Ac-ILE and Ac-MYN were also observed to undergo crystallisation, but it was followed by fast fibrillisation (within a minute), indicating a nanocrystalline origin for their self-assembly. From the series of tripeptide concentrations (5–40 mg/mL), the dependence of phase transition (crystal-to-fibril^82^) on peptide concentration is apparent. However, while the crystal-to-fibril transition was spontaneous for Ac-L^*n*^VD, Ac-VLE, Ac-ILE, the crystallites of Ac-MYN were stable enough that prolonged sonication has to be applied to induce fibrillisation of Ac-MYN. Presumably, sonication imparts the energy required to induce a twist in the long axis of the crystallite, leading to a transition to fibrillar structure^83^. A nanocrystalline origin of fibrils for the tripeptides would be consistent with the myriad effects the various moiety variations exert on self-assembly, nanostructure formation, and phase transition. As different residue combinations would engender different intermolecular interactions, any small structural change would therefore affect the ordered packing of peptide. The outcome of an otherwise random peptide aggregation would not be so heavily dependent on the moiety variations we have examined. This is also consistent with the nucleation model proposed for protein and peptide aggregation^84^. While it might therefore be tempting to infer peptide packing in fibrils to crystalline structures, studies have shown that such comparisions have to be done cautiously^67,85–87^. However, it is also surprising that microbead formation is also affected by moiety variation. This is best exemplified by comparing the analogs of Ac-LLE and Ac-LVE: the analogs of Ac-LLE can generally form microbeads while those of Ac-LVE do not (Table 1). The difference that Tyr → Phe makes at P1 and P2 of Ac-YYD also illustrates the sensitivity of microbead formation to tripeptide structure. Thus, these results suggest that there could also be a nanocrystalline origin to microbead formation. Indeed, a high order of organisation has been determined for the “peptide beads” formed by undecapeptides^88^.

## Conclusion

It is certainly tempting and desirable to try to correlate changes in peptide self-assembled nanostructures to molecular moiety changes on the peptides. However, it has turned out that this is not a trivial correlation to make as no clear pattern has emerged from this first study, and it is apparent that more detailed and focussed studies are required to reveal such a correlation. Nonetheless, the structural information we have uncovered for Ac-LLE and Ac-YLD, as well as the self-assembly outcomes due to the single moiety changes to various tripeptides, can potentially be used to provide vital data to design and assemble novel peptide nanostructures. These novel peptide nanostructures can then be assessed under physiological conditions for their applicability in diverse biomedical applications^89^.

## Materials and Methods

All peptides are prepared via standard solid-phase peptide synthesis using fluoren-9-ylmethoxycarbonyl (Fmoc)-protected amino acids^90^. The Fmoc-protected amino acids, *O*-(benzotriazol-1-yl)-*N*,*N*,*N’*,*N’*-tetramethyluronium tetrafluoroborate (TBTU), and Rink/Wang resins used were purchased from GL Biochem (Shanghai). All other reagents, i.e. acetic anhydride, diisopropylethylamine, *N*,*N*-dimethylformamide, trifluoroacetic acid, triisopropylsilane, were purchased from Sigma-Aldrich. The peptides were purified via reverse phase high performance liquid chromatography and analyzed by mass spectrometry (HPLC-MS) on an Agilent 6130 Quadrupole LC/MS system using a gradient of acetonitrile/water and formic acid (0.1%). The molecular mass of each peptide was set as the trigger for fraction collection so that only the peptide was collected. The volume of the pooled fractions (of pure peptide) was reduced on a rotary evaporator, after which the remaining solution was flash-frozen at -78 °C and lyophilised on a Labconco freeze dryer under high vacuum. The purified peptides were then weighed out in 2 mL vials, after which water (0.5 mL) was added. The peptide-water mixtures were shaken and sonicated in an Elmasonic S60 sonicator (37 kHz, 150 W) at 25 °C for 30 s to dissolve/disperse the peptide evenly in the water. There is no significant increase in sonicator bath water temperature within 30 s. The vials were then allowed to stand undisturbed at 25 °C and observed over the course of two weeks. Analysis was carried either when a change in state was observed or after two weeks (when no more change was observed). It is only Ac-LLE for which a change in state (crystallisation) was observed only after two months.

### Field emission scanning electron microscopy (FESEM) Study of Peptide Morphology

For samples with precipitate, the mixture was centrifuged and the supernatant removed with a Pasteur pipette. This would allow the determination of the structures that can co-exist with each other in the two separate phases. The supernatant was then filtered through a small cotton bud to remove any residual precipitate. The solution (or supernatant), precipitate, or hydrogel was flash-frozen at -78 °C, after which the frozen sample was lyophilised on a freeze dryer. A thin layer of the dried peptide sample was then spread on copper conductive tape, and a thin layer of platinum was sputter-coated on the peptide in a JEOL JFC-1600 Auto Fine Coater. The morphology of the peptide was analyzed with a JEOL JSM-7400F field emission scanning electron microscope. The analyses were carried out in a vacuum (10 Pa) using a current of 10 A (5 kV) and a working distance of 8–9 mm. The presence of amorphous, bead, or crystalline structures can be discerned at 1000×, but fibrillar nanostructures can only be discerned at >10000×.

### X-ray crystallographic Study of Ac-LLE

Crystallisation and structure solution of Ac-LLE followed the procedure reported for Ac-YLD^60^. Briefly, Ac-LLE in a glass vial was dissolved in water to 30 mg/mL, and was allowed to crystallise spontaneously at ambient temperature over three months. The crystals were then transferred into 25% (v/v) glycerol for five minutes before being flash frozen in liquid nitrogen. X-ray diffraction data were collected at 100 K on a Bruker × 8 PROTEUM system consisting of a MICROSTAR micro-focus X-ray generator, a PLATINUM135 CCD detector, and a 4-circle KAPPA goniometer. Data reduction was carried out using SAINT, SADABS, and XPREP, which are part of the Bruker Proteum2 program suite^91^. Ab initio structural determination was achieved using SHELXD^92^, and the model obtained was further refined using SHELXL^93^ through the ShelXle graphic user interface^94^. Coot^95^ and The PyMOL Molecular Graphics System (Version 1.7.4 Schrödinger, LLC.) were used to visualise the structure and generate figures. Details of crystallisation, data collection and refinement are listed in Supplementary Table S1, which was generated with publCIF^96^. The final structure has been deposited at the Cambridge Crystallographic Data Centre with the deposition number CCDC 1411702.

## Electronic supplementary material

CIF check file

Supplementary information
