# Supplementary material for: Systematic Moiety Variations of Ultrashort Peptides Produce Profound Effects on Self-Assembly, Nanostructure Formation, Hydrogelation, and Phase Transition
Source: Sci Rep. 2017 Oct 10;7:12897. doi: 10.1038/s41598-017-12694-9 (PMC5635115; doi:10.1038/s41598-017-12694-9)

checkCIF/PLATON report

Structure factors have been supplied for datablock(s) redo2

THIS REPORT IS FOR GUIDANCE ONLY. IF USED AS PART OF A REVIEW PROCEDURE FOR PUBLICATION, IT SHOULD NOT REPLACE THE EXPERTISE OF AN EXPERIENCED CRYSTALLOGRAPHIC REFEREE.

No syntax errors found. CIF dictionary Interpreting this report

Datablock: redo2

```
Bond precision:  C-C = 0.0112 A         Wavelength=1.54178

Cell:           a=31.362(3)    b=5.4514(5)         c=13.3403(13)
                alpha=90       beta=102.504(5)     gamma=90
Temperature:    100 K

                Calculated                 Reported
Volume          2226.7(4)                  2226.7(4)
Space group     C 2                        C 2
Hall group      C 2y                       C 2y
Moiety formula  C19 H33 N3 O7              ?
Sum formula     C19 H33 N3 O7              C19 H33 N3 O7
Mr              415.48                     415.48
Dx,g cm-3       1.239                      1.239
Z               4                          4
Mu (mm-1)       0.786                      0.786
F000            896.0                      896.0
F000'           898.98
h,k,lmax        32,5,14                    32,5,14
Nref            2711[ 1533]                2662
Tmin,Tmax       0.927,0.969
Tmin'           0.924

Correction method= Not given

Data completeness= 1.74/0.98      Theta(max)= 54.225

R(reflections)= 0.0588( 1785)     wR2(reflections)= 0.1662( 2662)

S = 0.942                 Npar= 273
```

The following ALERTS were generated. Each ALERT has the format
test-name_ALERT_alert-type_alert-level.
Click on the hyperlinks for more details of the test.

Alert level A

```
THETM01_ALERT_3_A  The value of sine(theta_max)/wavelength is less than 0.550
            Calculated sin(theta_max)/wavelength =    0.5262
```

Alert level B

```
PLAT340_ALERT_3_B Low Bond Precision on  C-C Bonds ...............     0.0112 Ang.
```

Alert level C

```
PLAT089_ALERT_3_C Poor Data / Parameter Ratio (Zmax < 18) ........       5.59 Note
PLAT222_ALERT_3_C Non-Solvent Resd 1   H Uiso(max)/Uiso(min) Range        4.3 Ratio
PLAT911_ALERT_3_C Missing # FCF Refl Between THmin & STh/L=  0.526          6 Report
PLAT978_ALERT_2_C Number C-C Bonds with Positive Residual Density.          0 Note
```

Alert level G

```
PLAT002_ALERT_2_G Number of Distance or Angle Restraints on AtSite         29 Note
PLAT007_ALERT_5_G Number of Unrefined Donor-H Atoms ..............          5 Report
PLAT032_ALERT_4_G Std. Uncertainty on Flack Parameter Value High .      0.400 Report
PLAT093_ALERT_1_G No s.u.'s on H-positions, Refinement Reported as      mixed Check
PLAT128_ALERT_4_G Alternate Setting for Input Space Group  C2              I2 Note
PLAT172_ALERT_4_G The CIF-Embedded .res File Contains DFIX Records         27 Report
PLAT173_ALERT_4_G The CIF-Embedded .res File Contains DANG Records         32 Report
PLAT174_ALERT_4_G The CIF-Embedded .res File Contains FLAT Records          4 Report
PLAT432_ALERT_2_G Short Inter X...Y Contact  OxT_13 ..  Ch3_10  ..       3.00 Ang.
PLAT720_ALERT_4_G Number of Unusual/Non-Standard Labels ..........         62 Note
PLAT791_ALERT_4_G The Model has Chirality at Ca_11   (Chiral SPGR)          S Verify
PLAT791_ALERT_4_G The Model has Chirality at Ca_12   (Chiral SPGR)          S Verify
PLAT791_ALERT_4_G The Model has Chirality at Ca_13   (Chiral SPGR)          S Verify
PLAT860_ALERT_3_G Number of Least-Squares Restraints .............         86 Note
PLAT916_ALERT_2_G Hooft y and Flack x Parameter values differ by .       0.18 Check
```

---

1 **ALERT level A** = Most likely a serious problem - resolve or explain
1 **ALERT level B** = A potentially serious problem, consider carefully
4 **ALERT level C** = Check. Ensure it is not caused by an omission or oversight
15 **ALERT level G** = General information/check it is not something unexpected

1 ALERT type 1 CIF construction/syntax error, inconsistent or missing data
4 ALERT type 2 Indicator that the structure model may be wrong or deficient
6 ALERT type 3 Indicator that the structure quality may be low
9 ALERT type 4 Improvement, methodology, query or suggestion
1 ALERT type 5 Informative message, check

It is advisable to attempt to resolve as many as possible of the alerts in all categories. Often the minor alerts point to easily fixed oversights, errors and omissions in your CIF or refinement strategy, so attention to these fine details can be worthwhile. In order to resolve some of the more serious problems it may be necessary to carry out additional measurements or structure refinements. However, the purpose of your study may justify the reported deviations and the more serious of these should normally be commented upon in the discussion or experimental section of a paper or in the "special_details" fields of the CIF. checkCIF was carefully designed to identify outliers and unusual parameters, but every test has its limitations and alerts that are not important in a particular case may appear. Conversely, the absence of alerts does not guarantee there are no aspects of the results needing attention. It is up to the individual to critically assess their own results and, if necessary, seek expert advice.

Publication of your CIF in IUCr journals

A basic structural check has been run on your CIF. These basic checks will be run on all CIFs submitted for publication in IUCr journals (*Acta Crystallographica*, *Journal of Applied Crystallography*, *Journal of Synchrotron Radiation*); however, if you intend to submit to *Acta Crystallographica Section C* or *E* or *IUCrData*, you should make sure that full publication checks are run on the final version of your CIF prior to submission.

Publication of your CIF in other journals

Please refer to the *Notes for Authors* of the relevant journal for any special instructions relating to CIF submission.

Validation response form

Please find below a validation response form (VRF) that can be filled in and pasted into your CIF.

```
# start Validation Reply Form
_vrf_THETM01_redo2
;
PROBLEM: The value of sine(theta_max)/wavelength is less than 0.550
RESPONSE: ...
;
_vrf_PLAT089_redo2
;
PROBLEM: Poor Data / Parameter Ratio (Zmax < 18) ........       5.59 Note
RESPONSE: ...
;
_vrf_PLAT222_redo2
;
PROBLEM: Non-Solvent Resd 1   H Uiso(max)/Uiso(min) Range        4.3 Ratio
RESPONSE: ...
;
_vrf_PLAT911_redo2
;
PROBLEM: Missing # FCF Refl Between THmin & STh/L=  0.526          6 Report
RESPONSE: ...
;
_vrf_PLAT978_redo2
;
PROBLEM: Number C-C Bonds with Positive Residual Density.          0 Note
RESPONSE: ...
;
# end Validation Reply Form
```

PLATON version of 27/03/2017; check.def file version of 24/03/2017

Datablock redo2 - ellipsoid plot

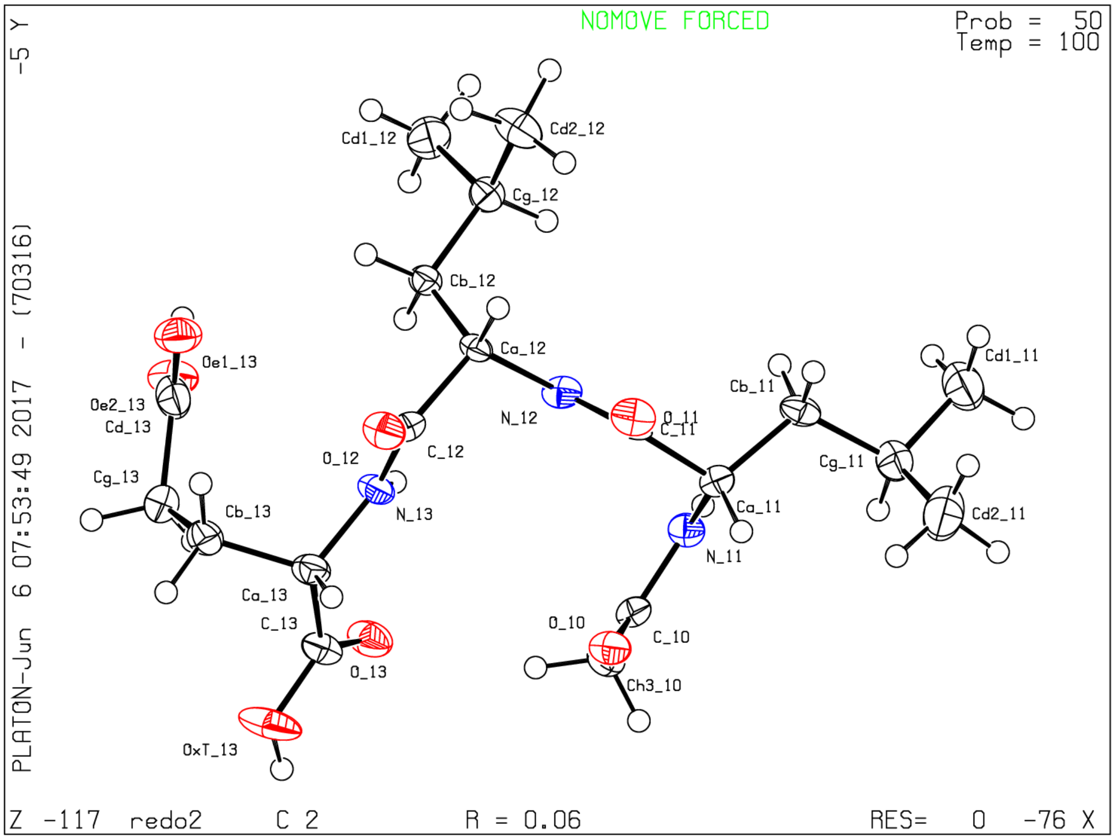

Supplement: Supplementary file 1 — CIF check file [file 41598_2017_12694_MOESM1_ESM.pdf]
