# Supplementary material for: Systematic Moiety Variations of Ultrashort Peptides Produce Profound Effects on Self-Assembly, Nanostructure Formation, Hydrogelation, and Phase Transition
Source: Sci Rep. 2017 Oct 10;7:12897. doi: 10.1038/s41598-017-12694-9 (PMC5635115; doi:10.1038/s41598-017-12694-9)
Supplement: Supplementary file 2 — Supplementary information [file 41598_2017_12694_MOESM2_ESM.pdf]

Supporting information for:

Systematic Moiety Variation of Ultrashort Peptides Produce Profound Effects on Self-Assembly, Nanostructure Formation, Hydrogelation, and Phase Transition

Kiat Hwa Chan[1,2]*, Bo Xue[3,4,5], Robert C. Robinson[3,5,6], and Charlotte A. E. Hauser[1,7]**

[1] Institute of Bioengineering and Nanotechnology, Biopolis, A*STAR (Agency for Science, Technology and Research), Singapore 138669
[2] Division of Science, Yale-NUS College, 16 College Avenue West, Singapore 138527
[3] Institute of Molecular and Cell Biology, Biopolis, A*STAR (Agency for Science, Technology and Research), Singapore 138673
[4] NUS Synthetic Biology for Clinical and Technological Innovation, Centre for Life Sciences, National University of Singapore, 28 Medical Drive, Singapore 117456
[5] Department of Biochemistry, Yong Loo Lin School of Medicine, National University of Singapore, 8 Medical Drive, Singapore 117597
[6] Research Institute for Interdisciplinary Science, Okayama University, Okayama 700-8530, Japan
[7] Laboratory for Nanomedicine, King Abdullah University of Science and Technology, Thuwal 23955-6900, Saudi Arabia

* kiathwa.chan@yale-nus.edu.sg
** charlotte.hauser@kaust.edu.sa

Contents 1

1. Crystalline aliphatic peptide 1: Ac-LLE 2

2. Crystalline aliphatic peptide 2: Ac-LVE 5

3. Crystalline aromatic peptide: Ac-YLD 8

4. Fibrillar aliphatic peptide 1: Ac-IVD 10

5. Fibrillar aliphatic peptide 2: Ac-VIE 12

6. Fibrilar aromatic peptide 1: Ac-MYD 15

7. Fibrillar aromatic peptide 2: Ac-YYD 18

8. Details of crystallisation, data collection and refinement 20

1. Crystalline Aliphatic Parent Tripeptide 1: Ac-LLE

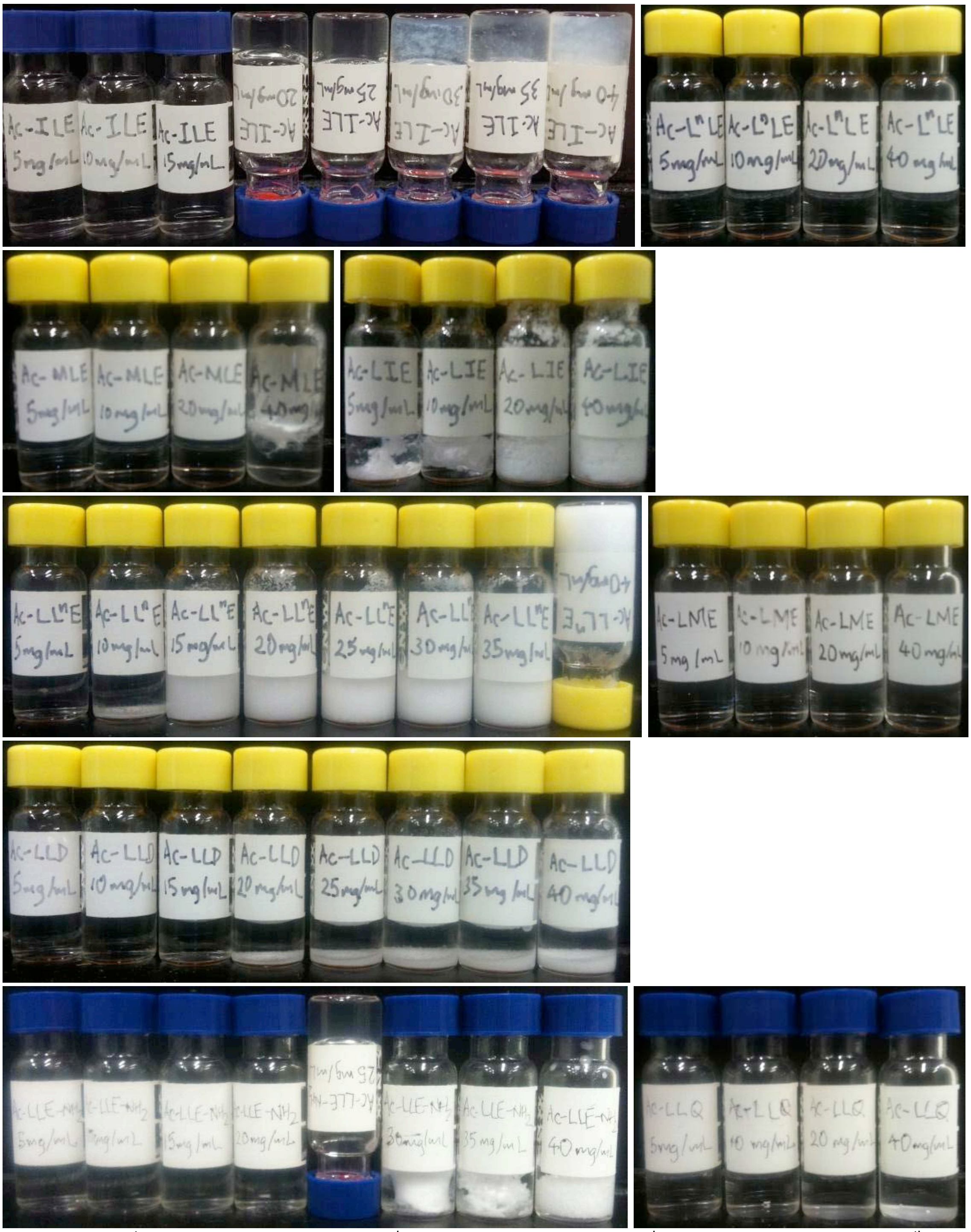

Figure S1. 1st row: Ac-ILE, Ac-L^{n}LE; 2nd row: Ac-MLE, Ac-LIE; 3rd row: Ac-LL^{n}E, Ac-LME; 4th row: Ac-LLD; 5th row: Ac-LLE-NH_2, Ac-LLQ.

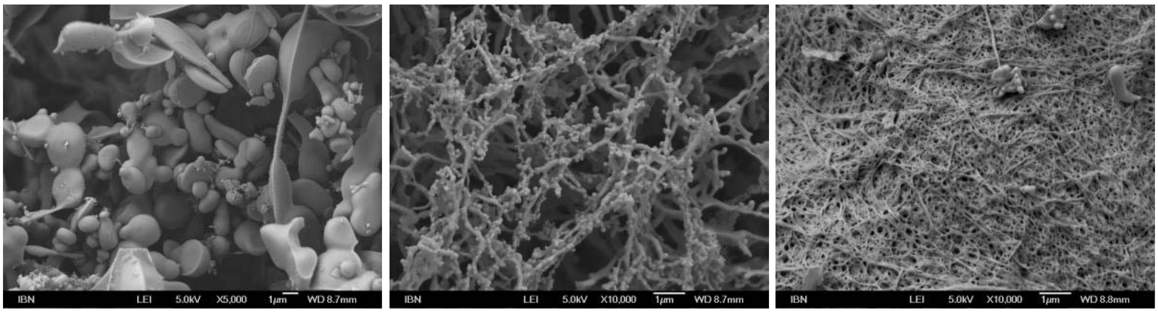

Figure S1-1. Electron micrographs of Ac-ILE: (Left) 5 mg/mL; (middle) 20 mg/mL; (right) 40 mg/mL

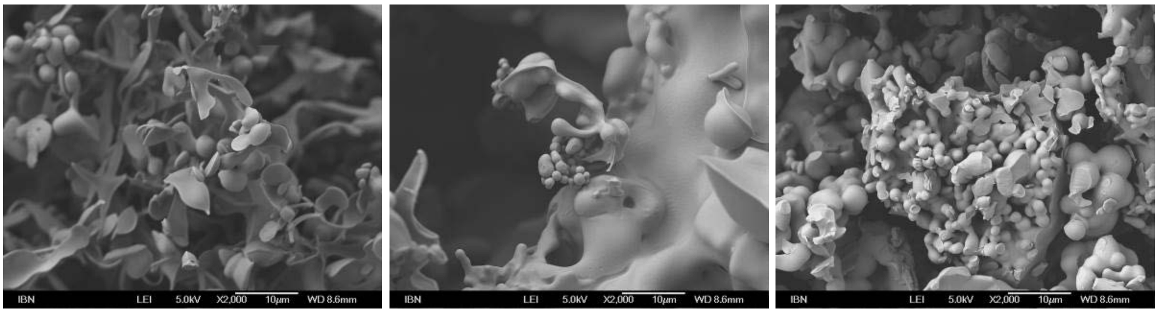

Figure S1-2. Electron micrographs of Ac-L^{n}LE: (Left) 5 mg/mL; (middle) 20 mg/mL; (right) 40 mg/mL

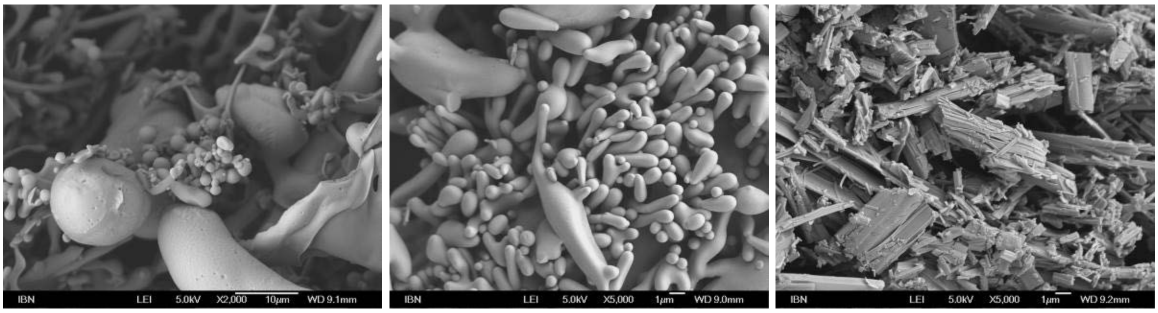

Figure S1-3. Electron micrographs of Ac-MLE: (Left) 5 mg/mL; (middle) 40 mg/mL, supernatant; (right) 40 mg/mL, precipitate (precipitation occurred after more than one month on standing)

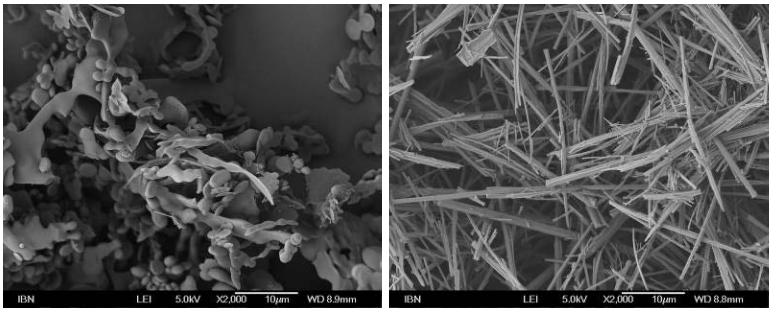

Figure S1-4. Electron micrographs of Ac-LIE: (Left) 5 mg/mL, supernatant; (middle) 5 mg/mL, precipitate; (right)

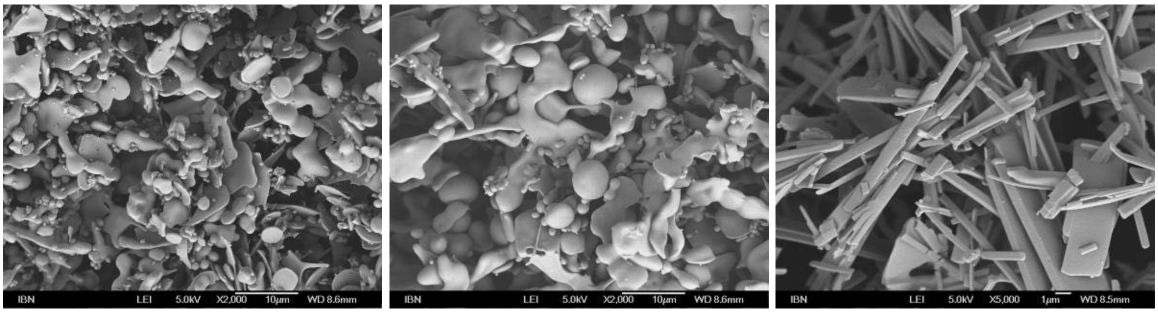

Figure S1-5. Electron micrographs of Ac-LL^{n}E: (Left) 5 mg/mL; (middle) 20 mg/mL, supernatant; (right) 20 mg/mL, precipitate

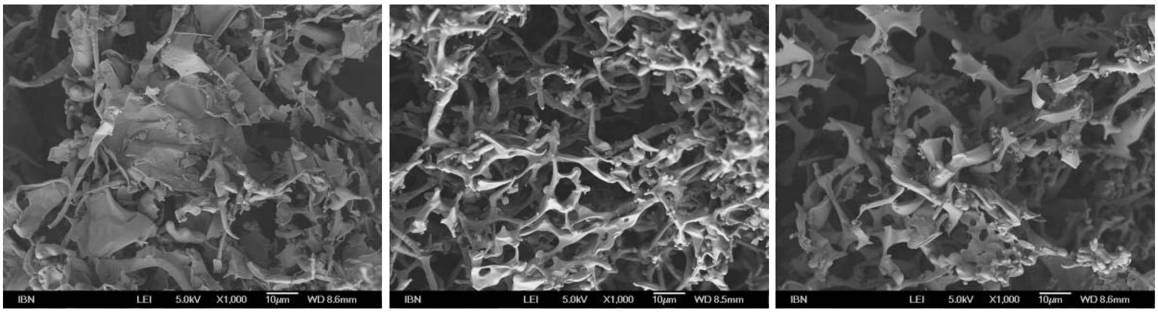

Figure S1-6. Electron micrographs of Ac-LME: (Left) 5 mg/mL; (middle) 20 mg/mL; (right) 40 mg/mL

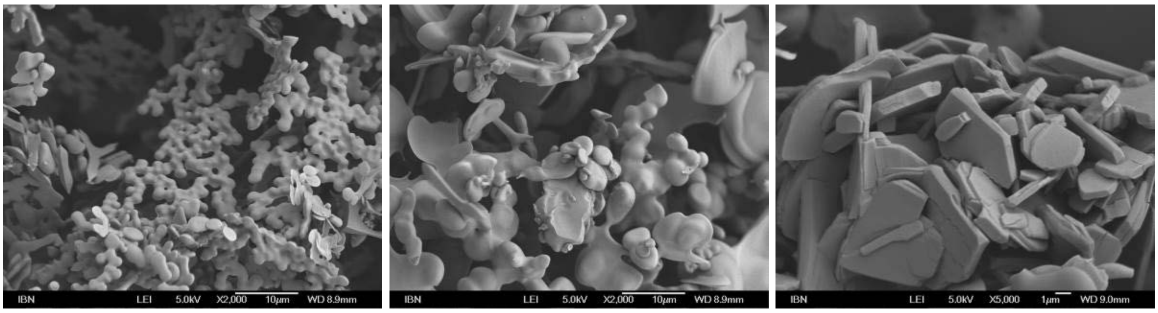

Figure S1-7. Electron micrographs of Ac-LLD: (Left) 5 mg/mL; (middle) 20 mg/mL, supernatant; (right) 20 mg/mL, precipitate

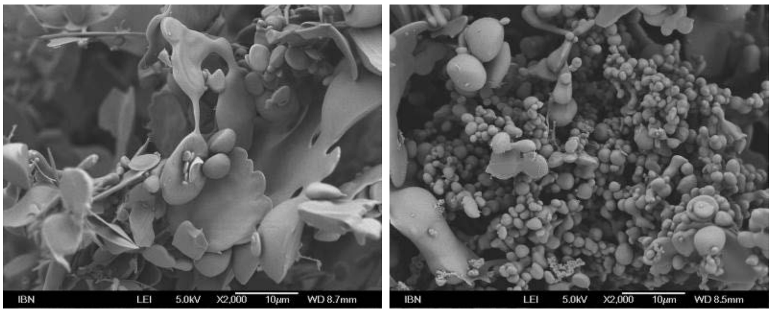

Figure S1-8. Electron micrographs of Ac-LLE-NH_2: (Left) 5 mg/mL; (right) 20 mg/mL

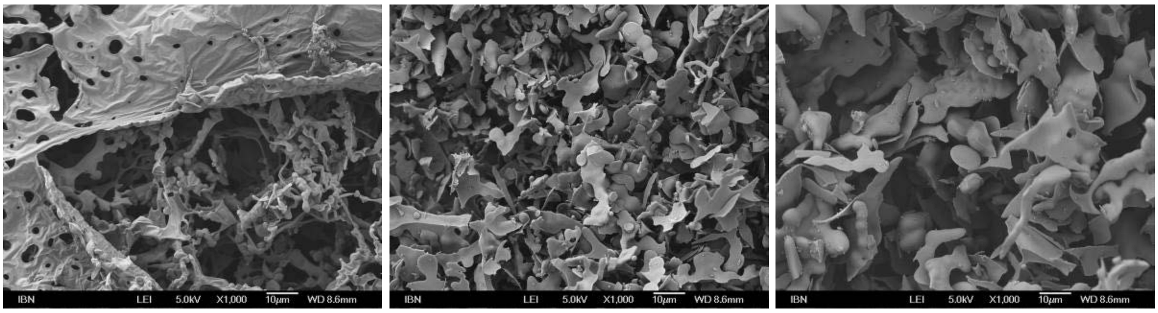

Figure S1-9. Electron micrographs of Ac-LLQ: (Left) 5 mg/mL; (middle) 20 mg/mL; (right) 40 mg/mL

2. Crystalline Aliphatic Parent Tripeptide 2: Ac-LVE

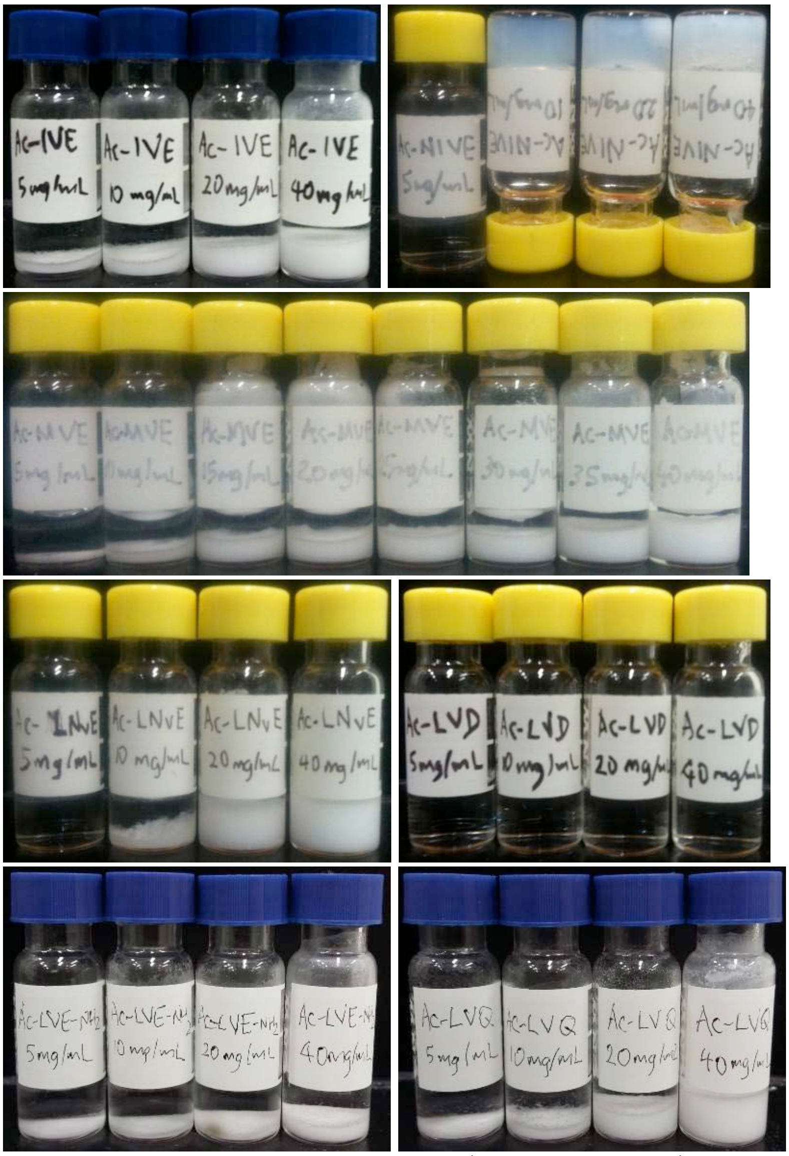

Figure S2. 1st row: Ac-IVE, Ac-L^{n}VE; 2nd row: Ac-MVE; 3rd row: Ac-LV^{n}E, Ac-LVD; 4th row: Ac-LVE-NH_2, Ac-LVQ.

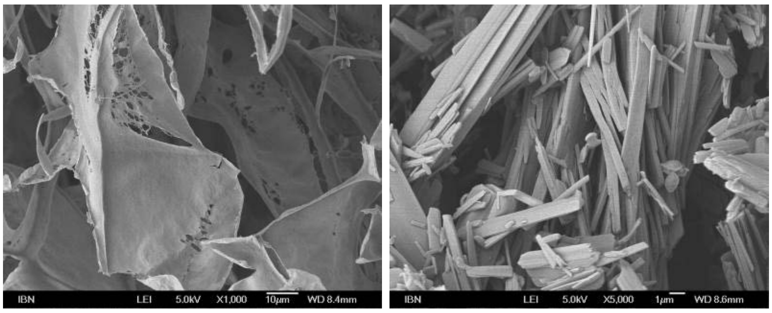

Figure S2-1. Electron micrographs of Ac-IVE: (Left) 5 mg/mL, supernatant; (right) 5 mg/mL, precipitate

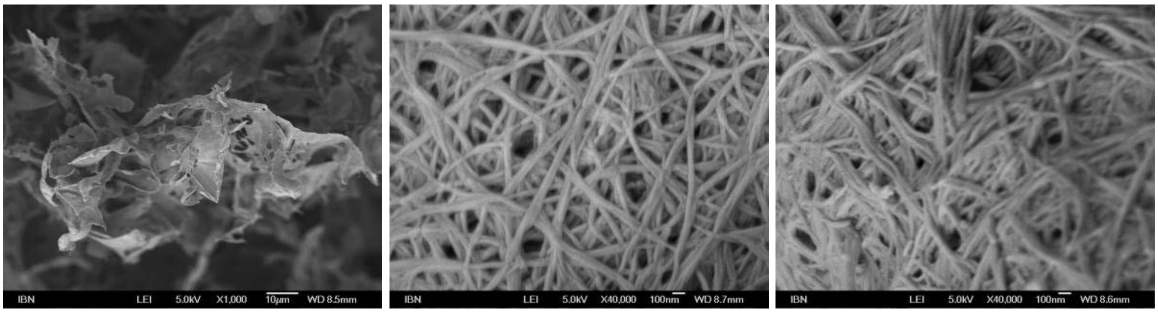

Figure S2-2. Electron micrographs of Ac-L^{n}VE: (Left) 5 mg/mL; (middle) 20 mg/mL; (right) 40 mg/mL

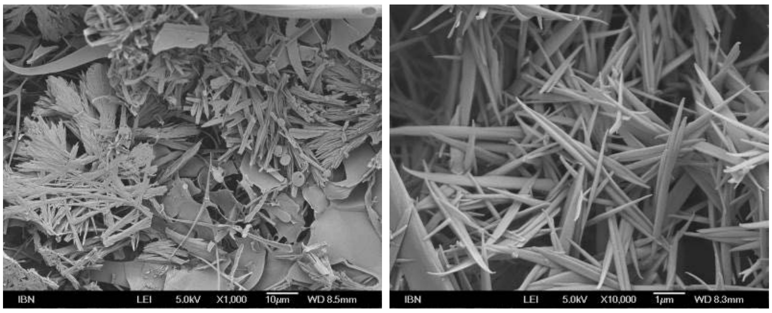

Figure S2-3. Electron micrographs of Ac-MVE: (Left) 5 mg/mL, supernatant; (right) 5 mg/mL, precipitate

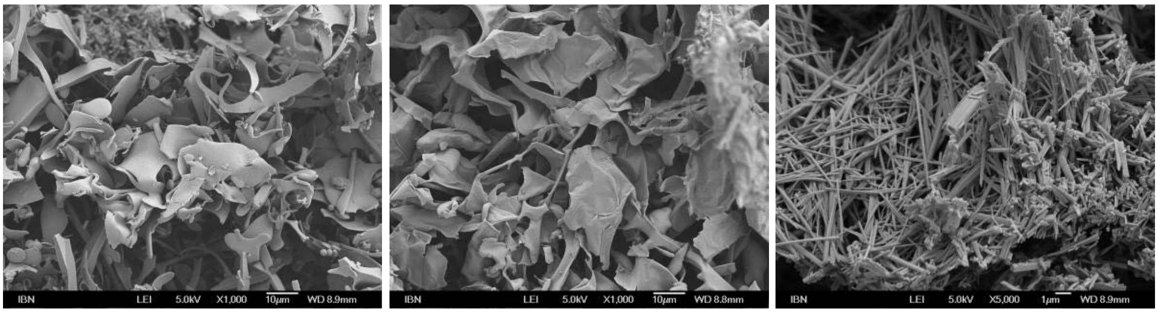

Figure S2-4. Electron micrographs of Ac-LV^{n}E: (Left) 5 mg/mL; (middle) 20 mg/mL, supernatant; (right) 20 mg/mL, precipitate (note biphasic distribution of crystallites and fibrils)

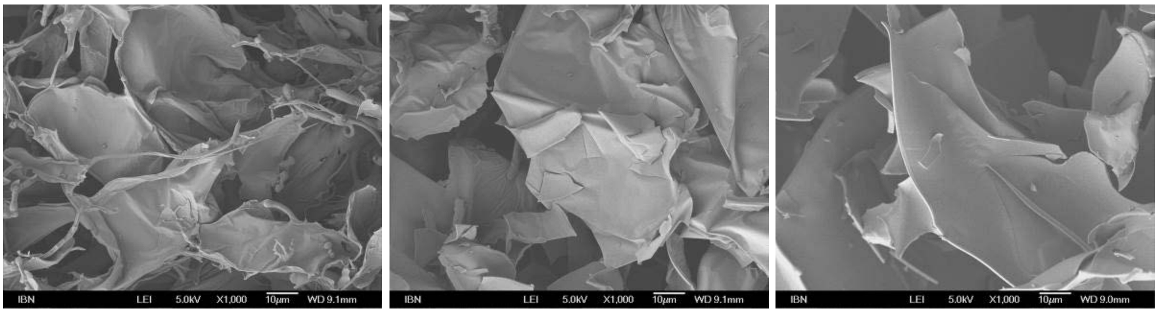

Figure S2-5. Electron micrographs of Ac-LVD: (Left) 5 mg/mL; (middle) 20 mg/mL; (right) 40 mg/mL

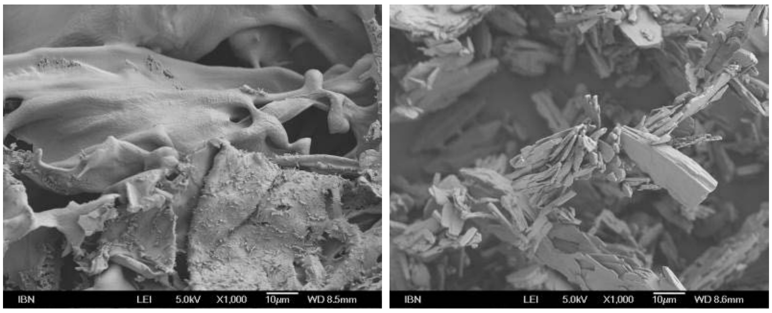

Figure S2-6. Electron micrographs of Ac-LVE-NH_2: (Left) 5 mg/mL, supernatant; (right) 5 mg/mL, precipitate

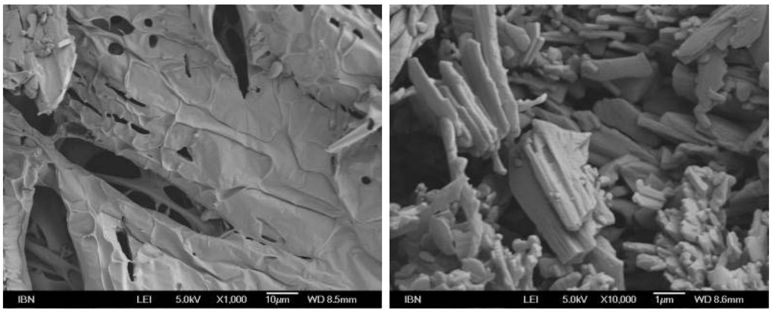

Figure S2-7. Electron micrographs of Ac-LVQ: (Left) 5 mg/mL, supernatant; (right) 5 mg/mL, precipitate

3. Crystalline Aromatic Parent Tripeptide: Ac-YLD

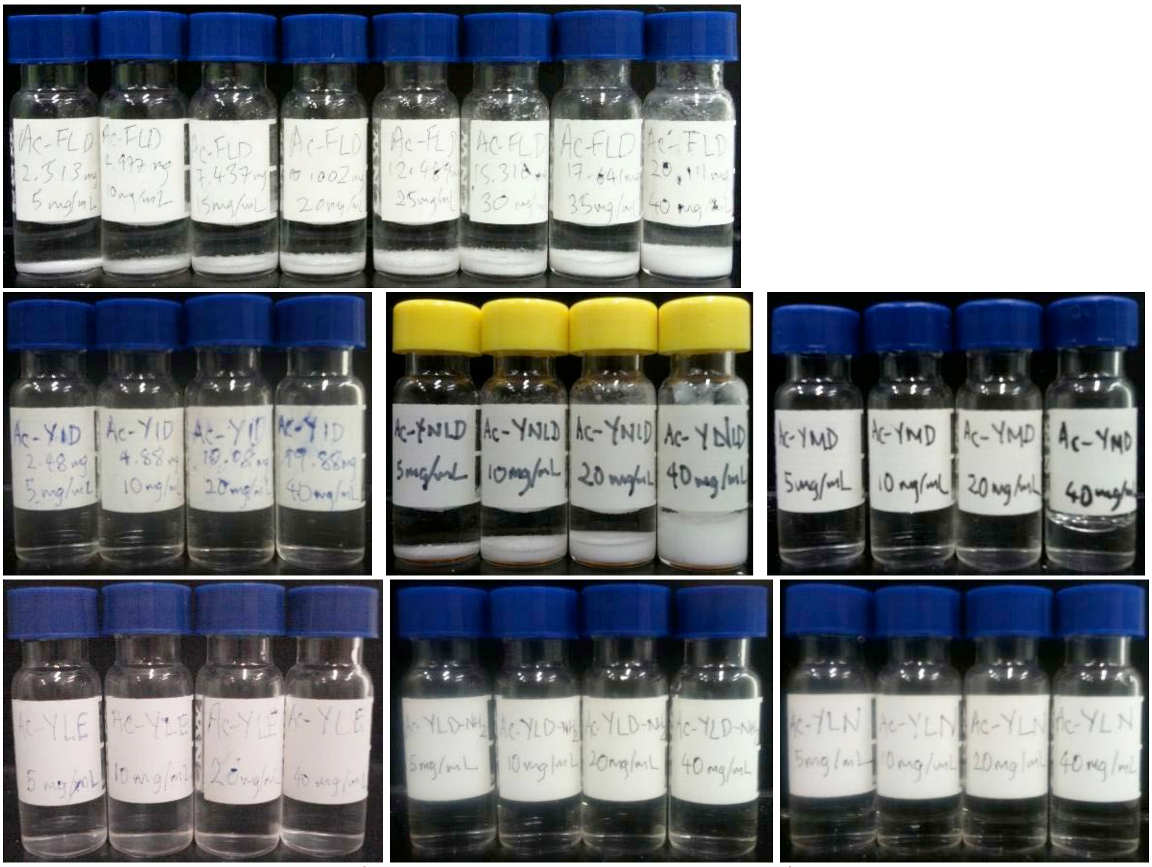

Figure S3. 1st row: Ac-FLD; 2nd row: Ac-YID, Ac-YL^{n}D, Ac-YMD; 3rd row: Ac-YLE, Ac-YLD-NH_2, Ac-YLN.

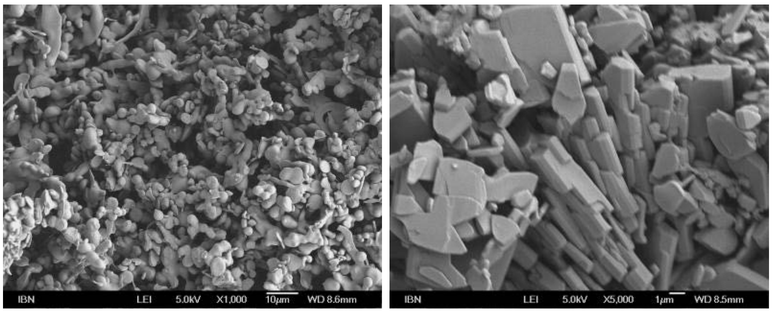

Figure S3-1. Electron micrographs of Ac-FLD: (Left) 5 mg/mL, supernatant; (right) 5 mg/mL, precipitate

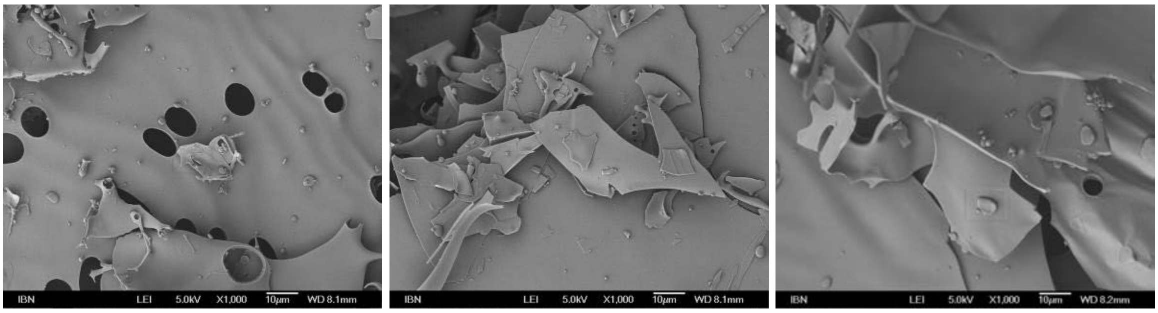

Figure S3-2. Electron micrographs of Ac-YID: (Left) 5 mg/mL; (middle) 20 mg/mL; (right) 40 mg/mL

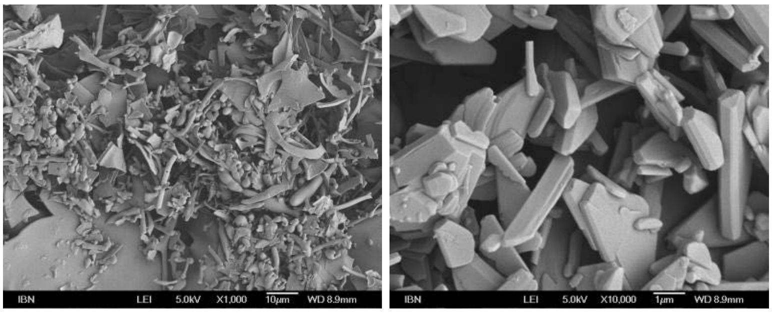

Figure S3-3. Electron micrographs of Ac-YL^{n}D: (Left) 5 mg/mL, supernatant; (right) 5 mg/mL, precipitate

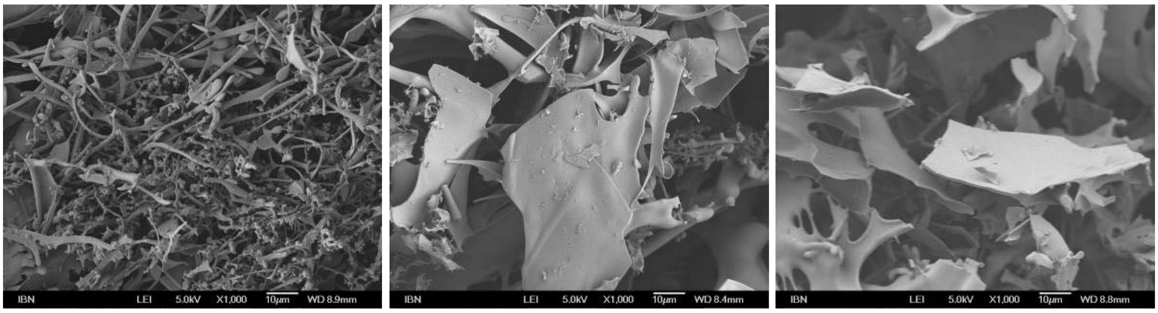

Figure S3-4. Electron micrographs of Ac-YMD: (Left) 5 mg/mL; (middle) 20 mg/mL; (right) 40 mg/mL

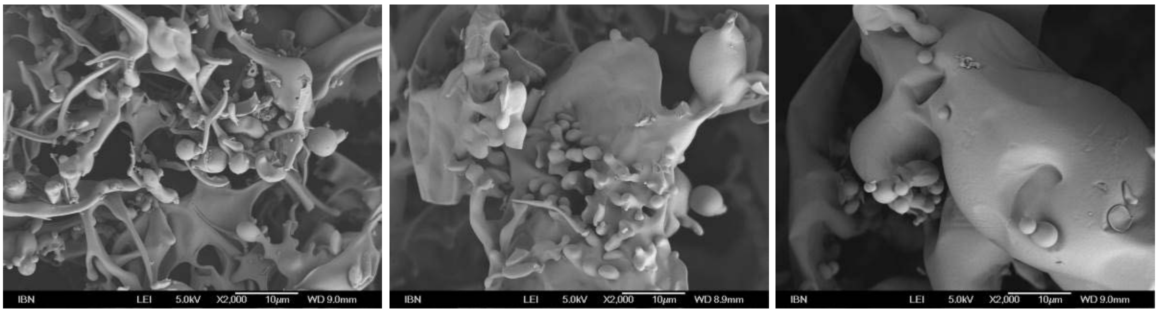

Figure S3-5. Electron micrographs of Ac-YLE: (Left) 5 mg/mL; (middle) 20 mg/mL; (right) 40 mg/mL

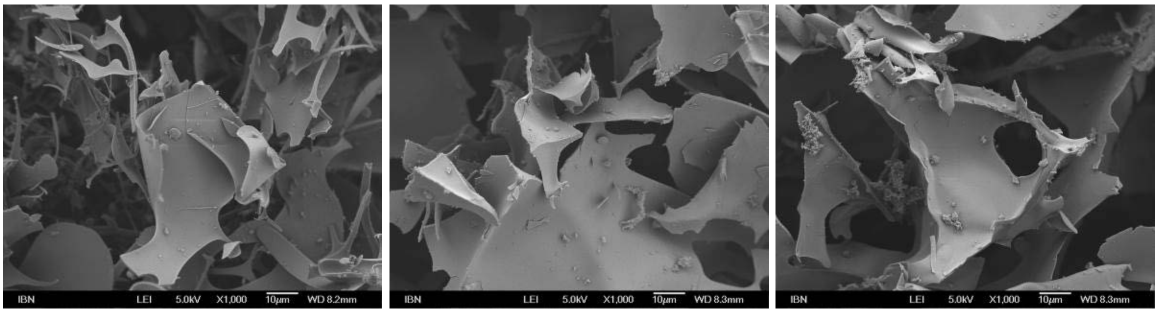

Figure S3-6. Electron micrographs of Ac-YLD-NH_2: (Left) 5 mg/mL; (middle) 20 mg/mL; (right) 40 mg/mL

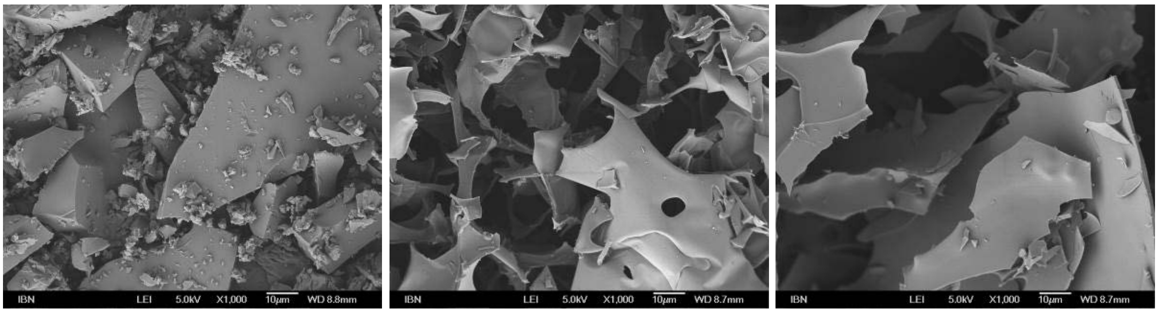

Figure S3-7. Electron micrographs of Ac-YLN: (Left) 5 mg/mL; (middle) 20 mg/mL; (right) 40 mg/mL

4. Fibrillar Aliphatic Parent Tripeptide 1: Ac-IVD

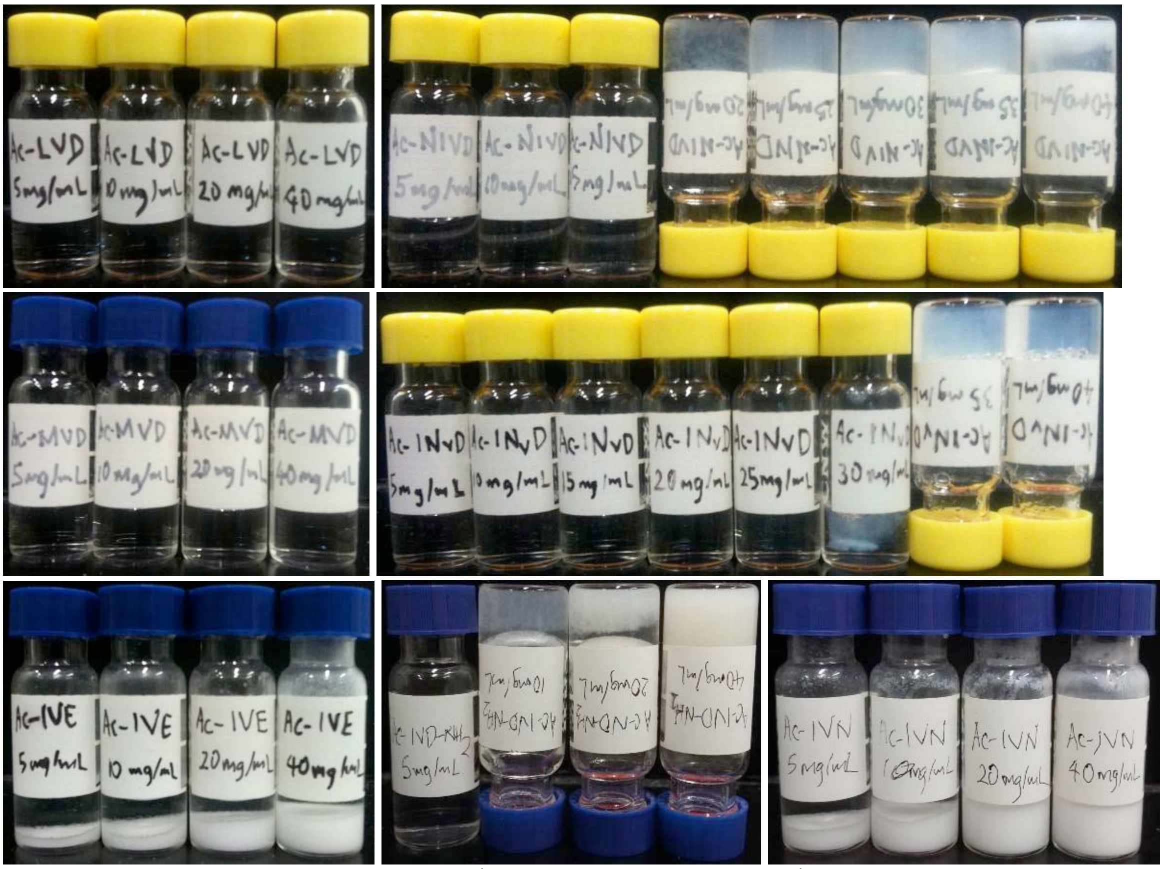

Figure S4. 1st row: Ac-LVD, Ac-L^{n}VD; 2nd row: Ac-MVD, Ac-IV^{n}D; 3rd row: Ac-IVE, Ac-IVD-NH_2, Ac-IVN.

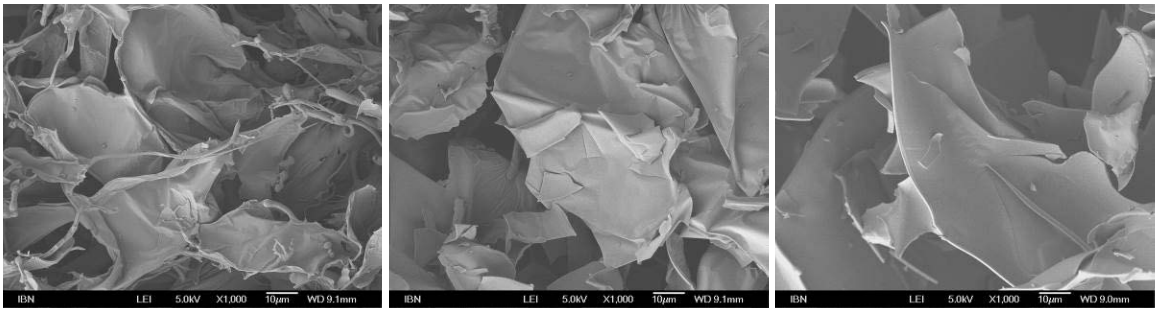

Figure S4-1. Electron micrographs of Ac-LVD: (Left) 5 mg/mL; (middle) 20 mg/mL; (right) 40 mg/mL

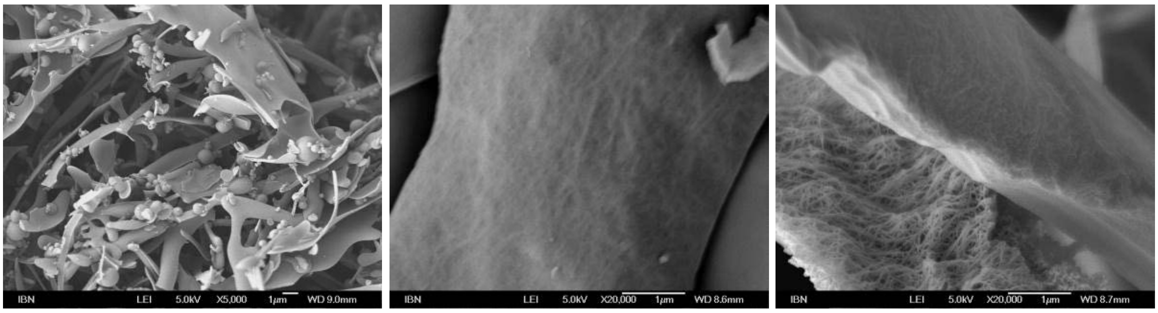

Figure S4-2. Electron micrographs of Ac-L^{n}VD: (Left) 5 mg/mL; (middle) 20 mg/mL; (right) 40 mg/mL

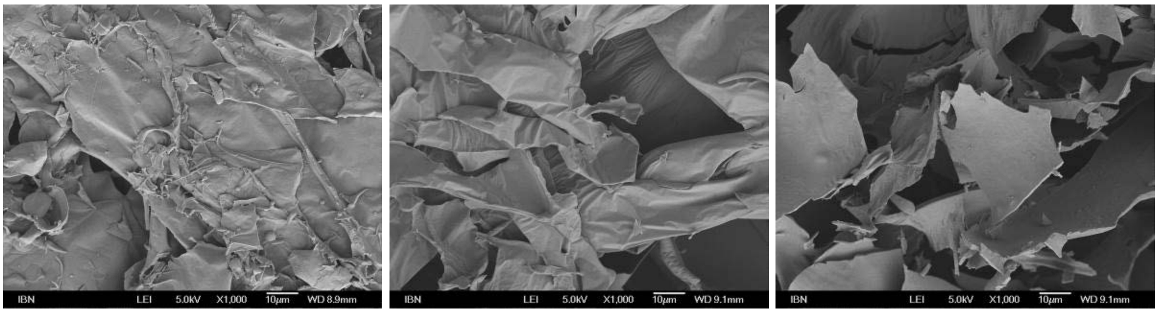

Figure S4-3. Electron micrographs of Ac-MVD: (Left) 5 mg/mL; (middle) 20 mg/mL; (right) 40 mg/mL

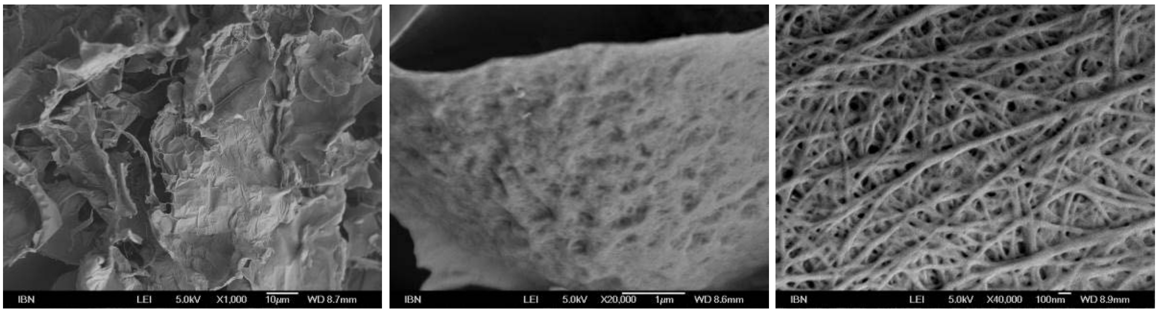

Figure S4-4. Electron micrographs of Ac-IV^{n}D: (Left) 5 mg/mL; (middle) 20 mg/mL; (right) 40 mg/mL

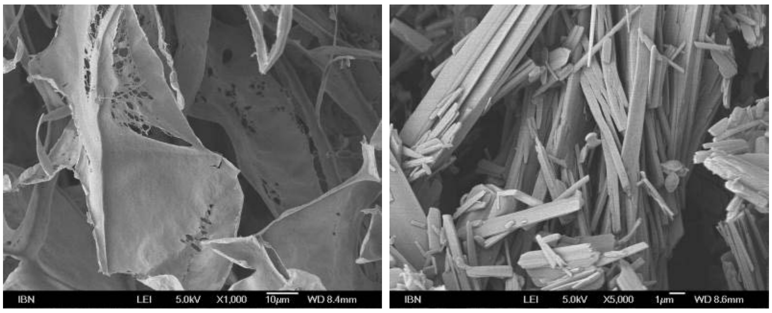

Figure S4-5. Electron micrographs of Ac-IVE: (Left) 5 mg/mL, supernatant; (right) 5 mg/mL, precipitate

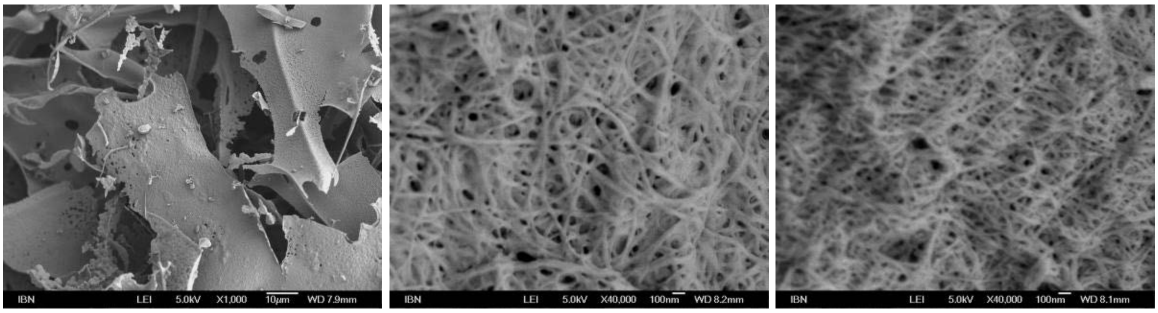

Figure S4-6. Electron micrographs of Ac-IVD-NH_2: (Left) 5 mg/mL; (middle) 20 mg/mL; (right) 40 mg/mL

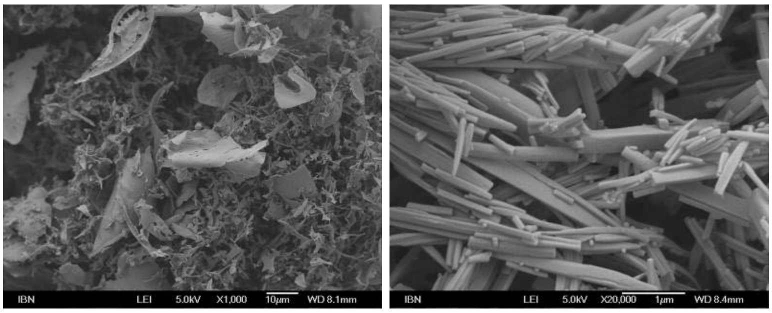

Figure S4-7. Electron micrographs of Ac-IVN: (Left) 5 mg/mL, supernatant; (right) 5 mg/mL, precipitate

5. Fibrillar Aliphatic Parent Tripeptide 2: Ac-VIE

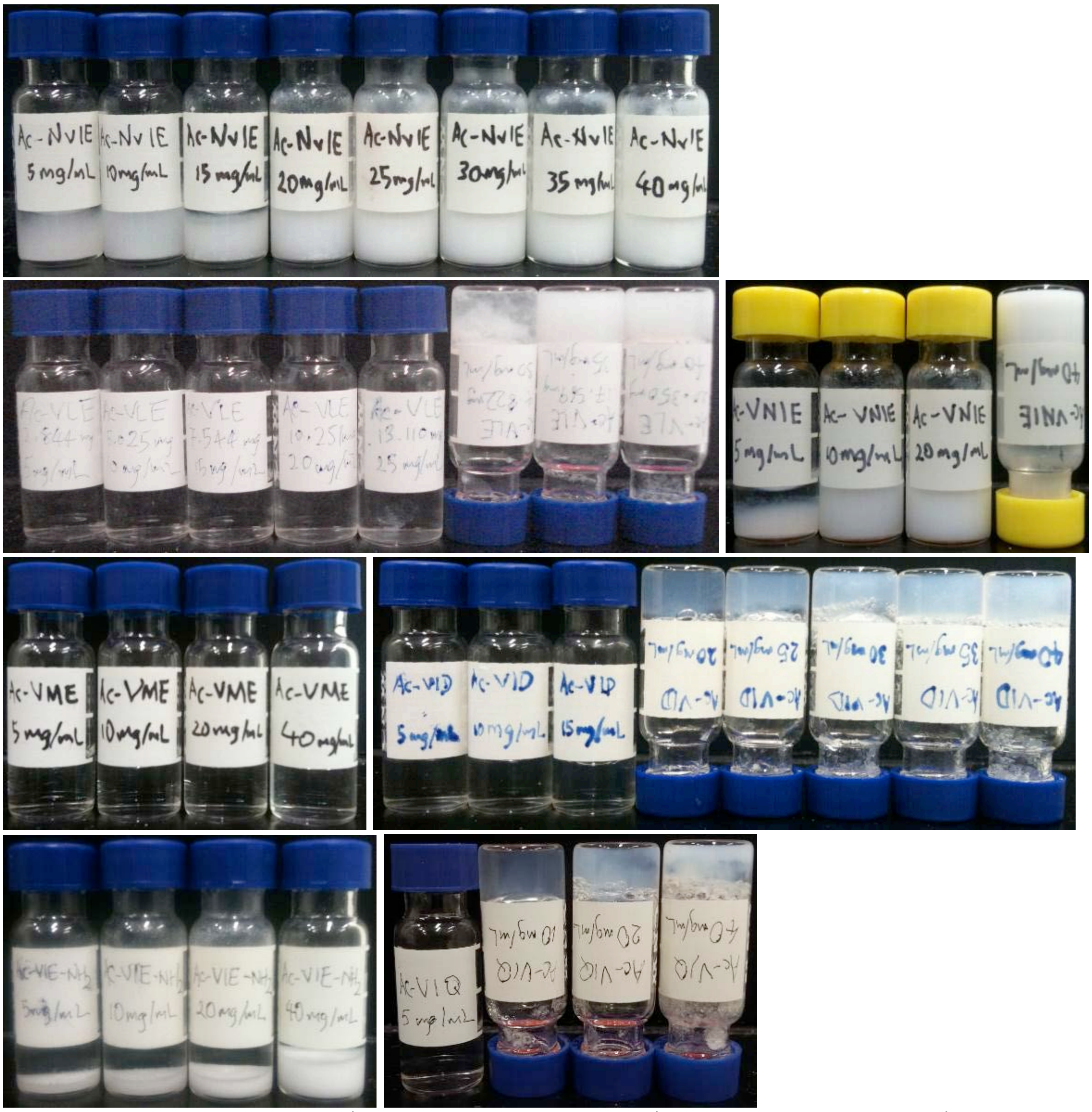
Figure S5. 1st row: Ac-V^{n}IE; 2nd row: Ac-VLE, Ac-VL^{n}E; 3rd row: Ac-VME, Ac-VID; 4th row: Ac-VIE-NH_2, Ac-VIQ.

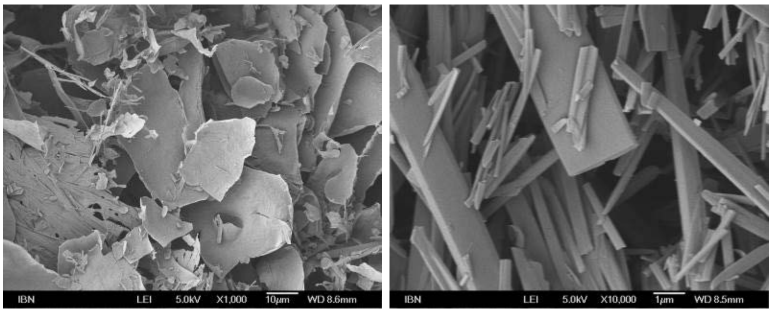

Figure S5-1. Electron micrographs of Ac-V^{n}IE: (Left) 5 mg/mL, supernatant; (right) 5 mg/mL, precipitate

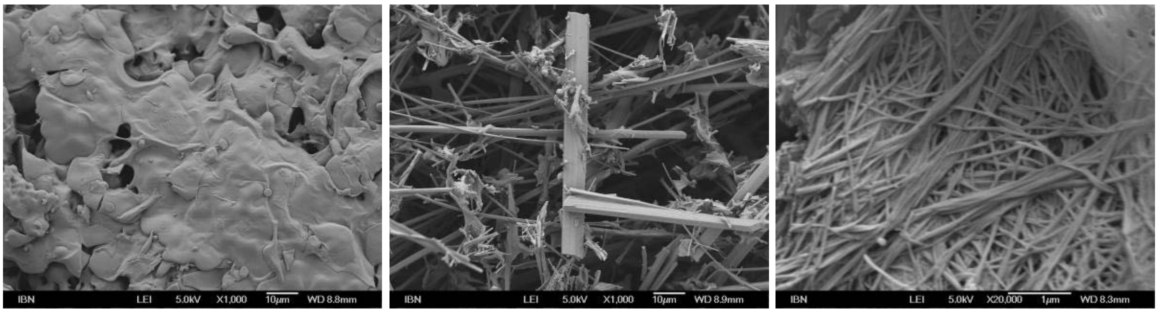

Figure S5-2. Electron micrographs of Ac-VLE: (Left) 20 mg/mL, supernatant; (middle) 20 mg/mL, precipitate; (right) 40 mg/mL

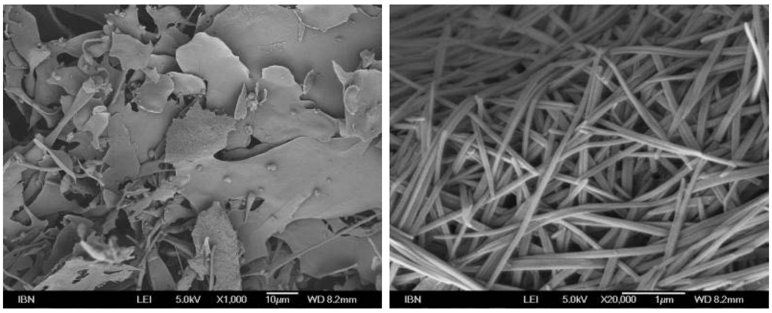

Figure S5-3. Electron micrographs of Ac-VL^{n}E: (Left) 5 mg/mL, supernatant; (right) 5 mg/mL, precipitate

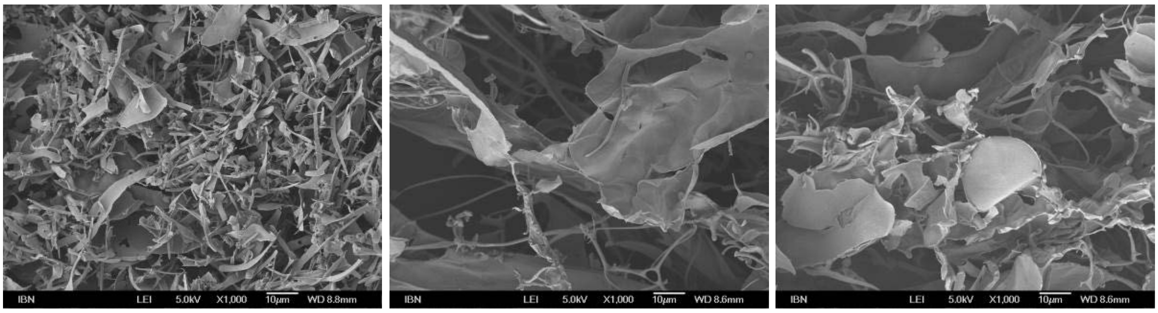

Figure S5-4. Electron micrographs of Ac-VME: (Left) 5 mg/mL; (middle) 20 mg/mL; (right) 40 mg/mL

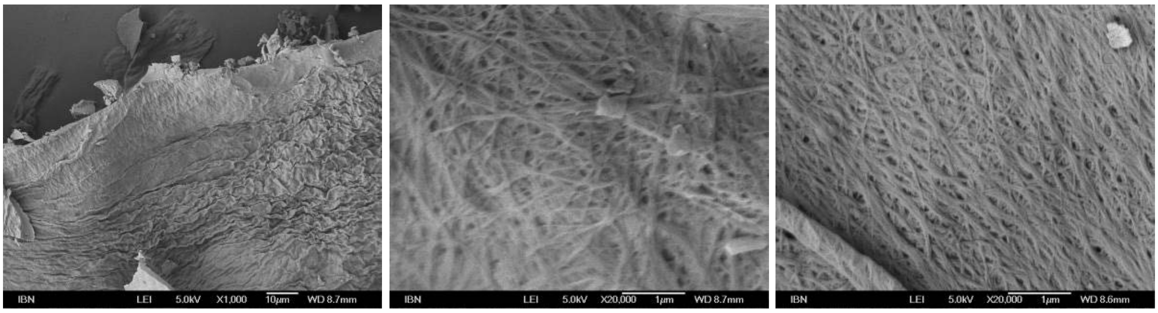

Figure S5-5. Electron micrographs of Ac-VID: (Left) 5 mg/mL; (middle) 20 mg/mL; (right) 40 mg/mL

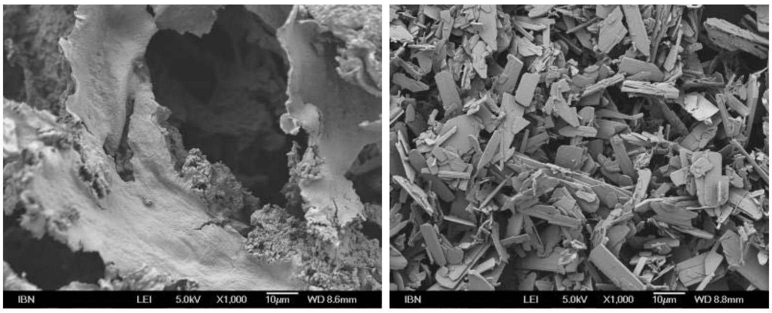

Figure S5-6. Electron micrographs of Ac-VIE-NH_2: (Left) 5 mg/mL, supernatant; (right) 5 mg/mL, precipitate

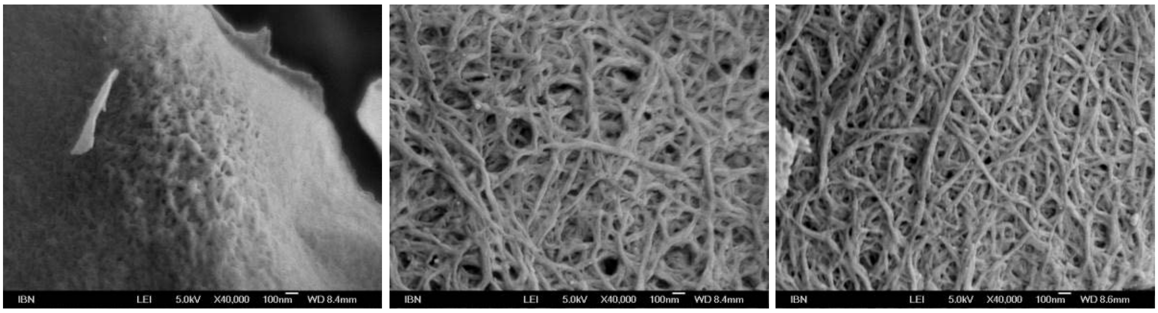

Figure S5-7. Electron micrographs of Ac-VIQ: (Left) 5 mg/mL; (middle) 20 mg/mL; (right) 40 mg/mL

6. Fibrillar Aromatic Parent Tripeptide 1: Ac-MYD

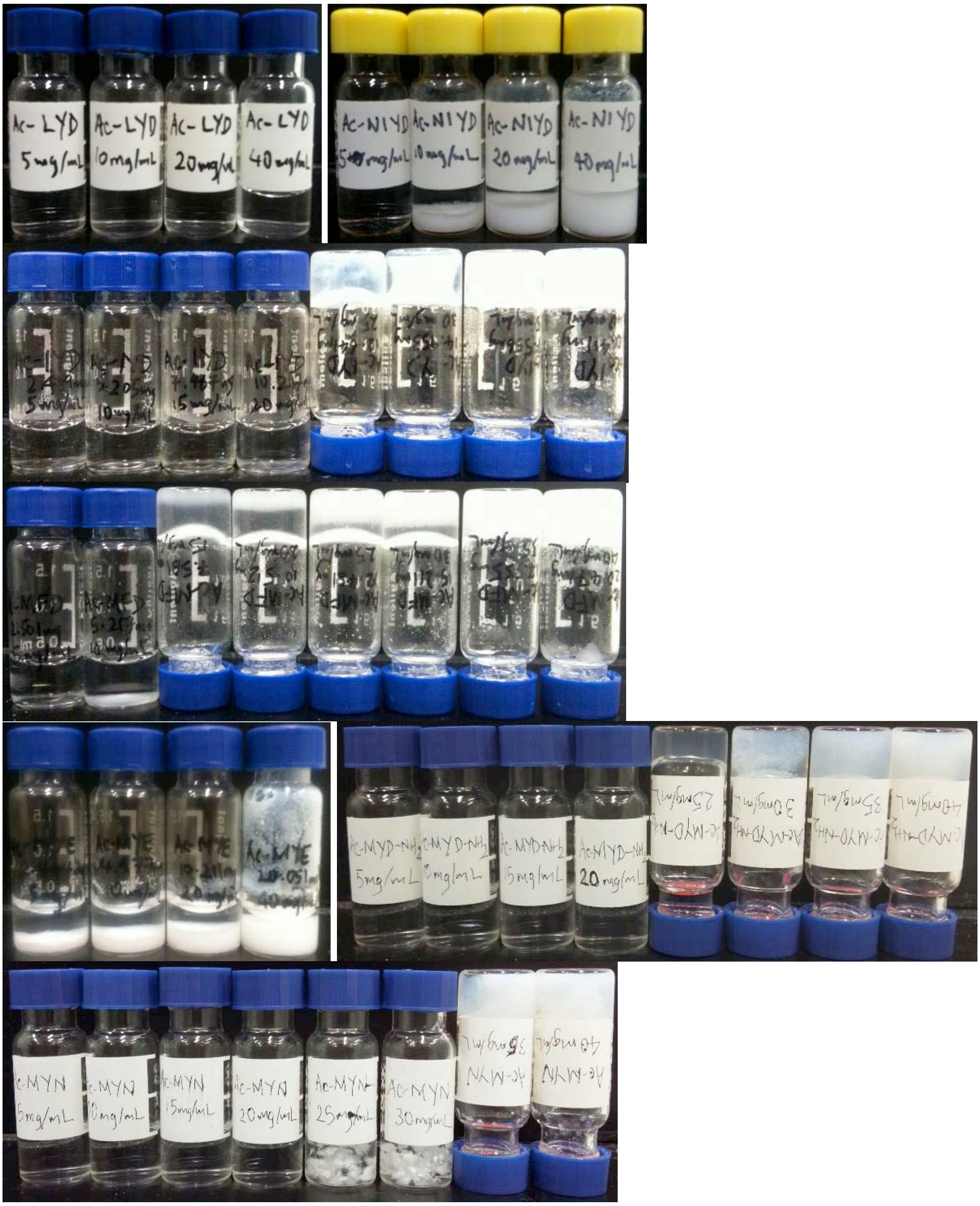

Figure S6. 1st row: Ac-IYD, Ac-LYD; 2nd row: Ac-L^{n}YD, Ac-MFD; 3rd row: Ac-MYE, Ac-MYD-NH$_2$; 4th row: Ac-MYN.

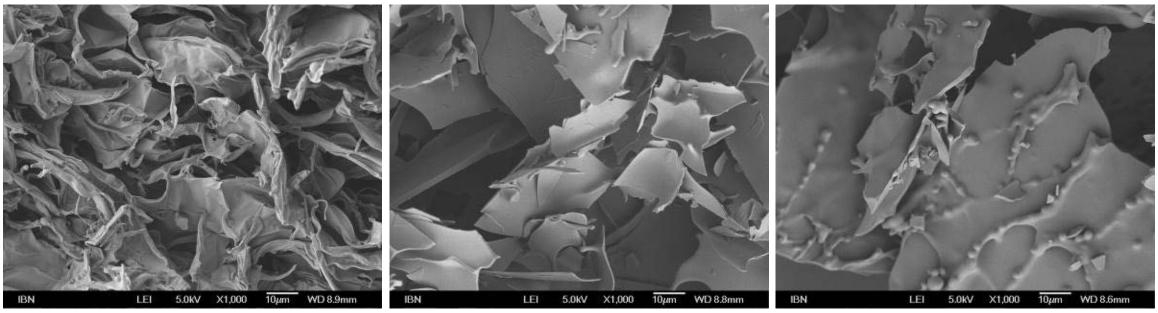

Figure S6-1. Electron micrographs of Ac-LYD: (Left) 5 mg/mL; (middle) 20 mg/mL; (right) 40 mg/mL

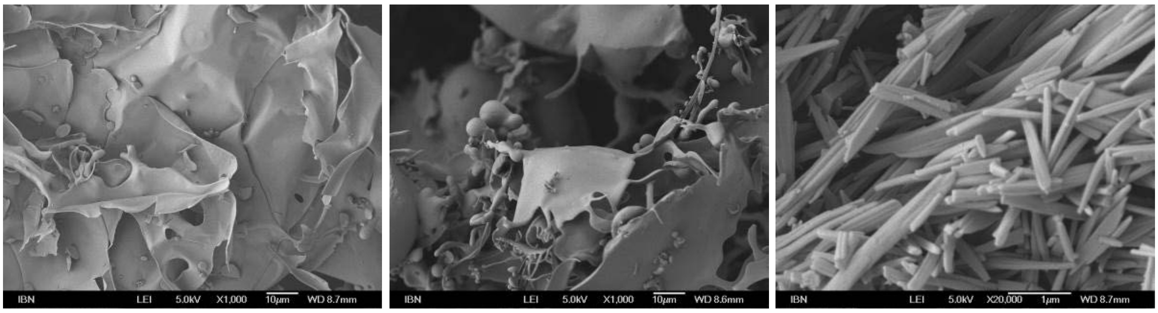

Figure S6-2. Electron micrographs of Ac-L^{n}YD: (Left) 5 mg/mL; (middle) 10 mg/mL, supernatant; (right) 10 mg/mL, precipitate

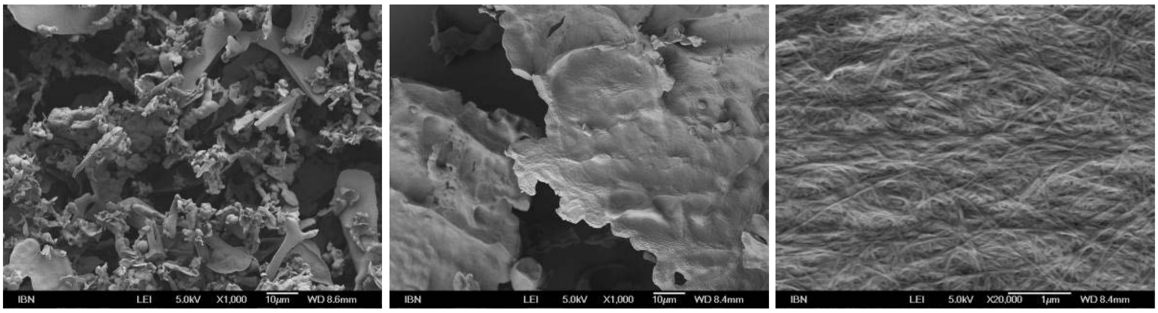

Figure S6-3. Electron micrographs of Ac-IYD: (Left) 5 mg/mL; (middle) 20 mg/mL, supernatant; (right) 20 mg/mL, precipitate

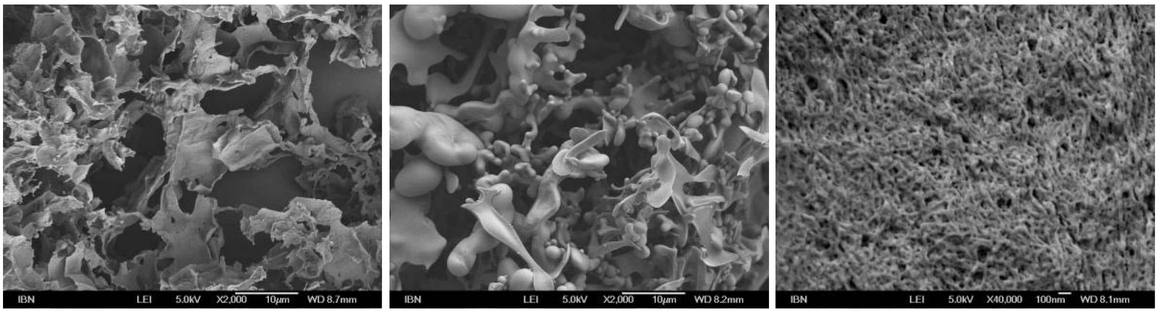

Figure S6-4. Electron micrographs of Ac-MFD: (Left) 5 mg/mL; (middle) 20 mg/mL, supernatant; (right) 20 mg/mL, precipitate

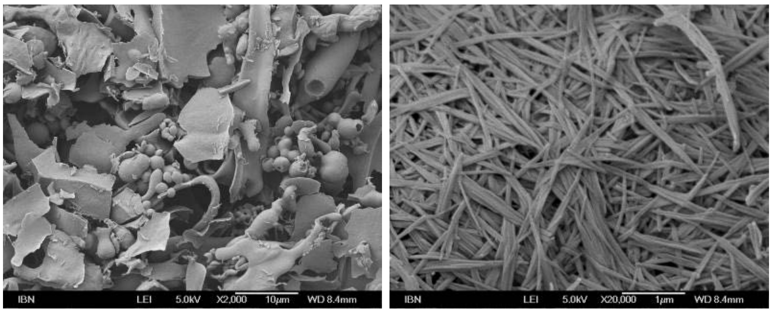

Figure S6-5. Electron micrographs of Ac-MYE: (Left) 5 mg/mL, supernatant; (right) 5 mg/mL, precipitate

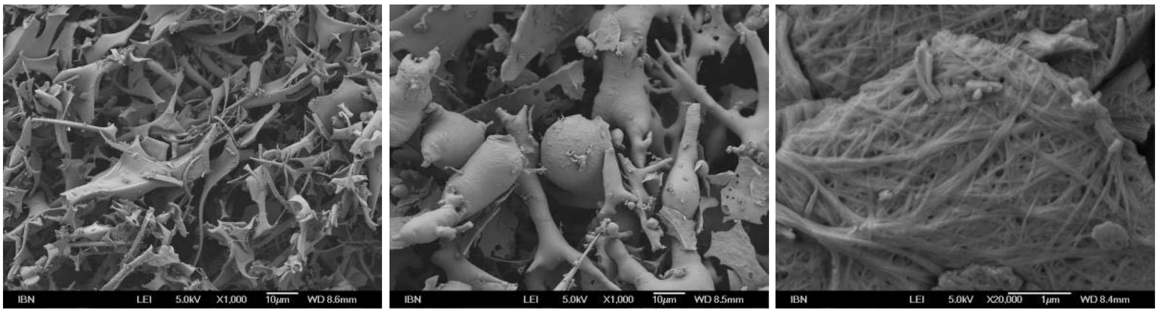

Figure S6-6. Electron micrographs of Ac-MYD-NH_2: (Left) 5 mg/mL; (middle) 20 mg/mL; (right) 40 mg/mL

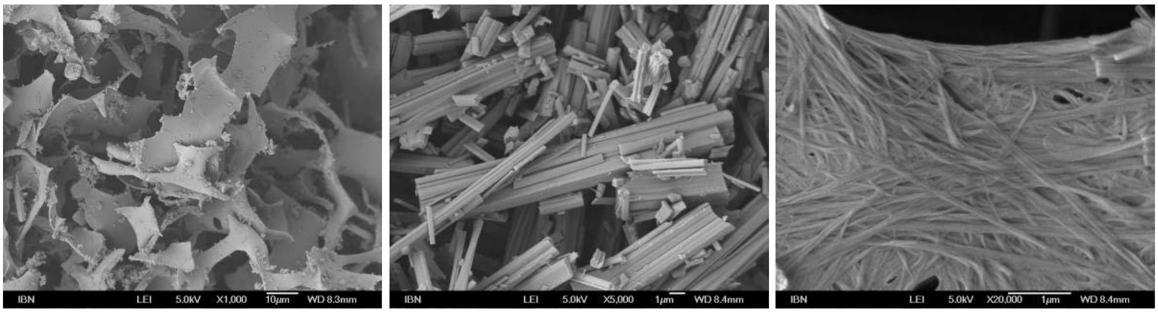

Figure S6-7. Electron micrographs of Ac-MYN: (Left) 40 mg/mL, supernatant; (middle) 40 mg/mL, precipitate (right) 40 mg/mL (which gelled after prolonged sonication)

7. Fibrillar Aromatic Parent Tripeptide 2: Ac-YYD

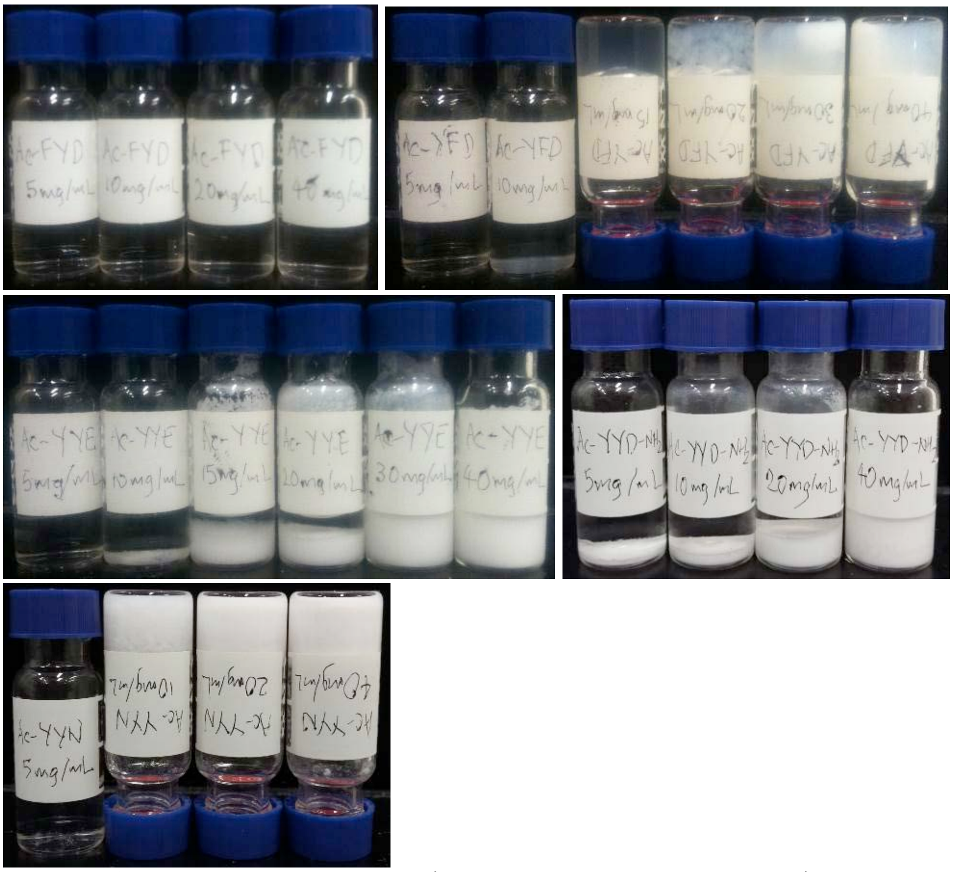

Figure. 1st row: Ac-FYD, Ac-YFD; 2nd row: Ac-YYE, Ac-YYD-NH_2; 3rd row: Ac-YYN.

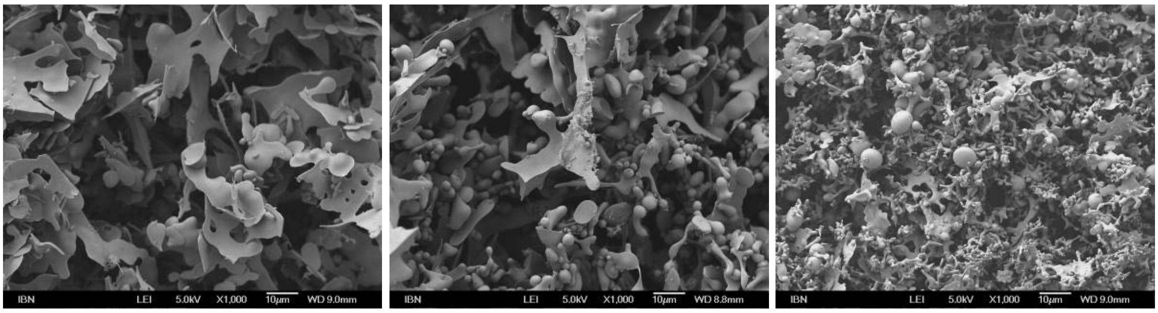

Figure S7-1. Electron micrographs of Ac-FYD: (Left) 5 mg/mL; (middle) 20 mg/mL; (right) 40 mg/mL

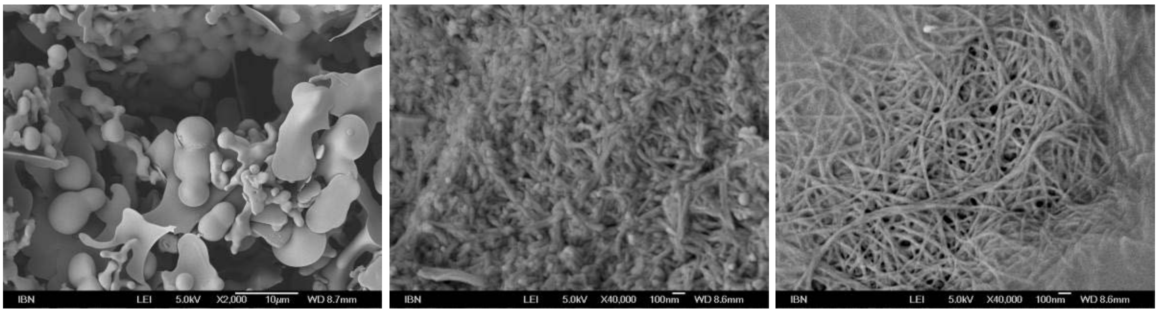

Figure S7-2. Electron micrographs of Ac-YFD: (Left) 10 mg/mL, supernatant; (middle) 10 mg/mL, hydrogel clumps; (right) 15 mg/mL

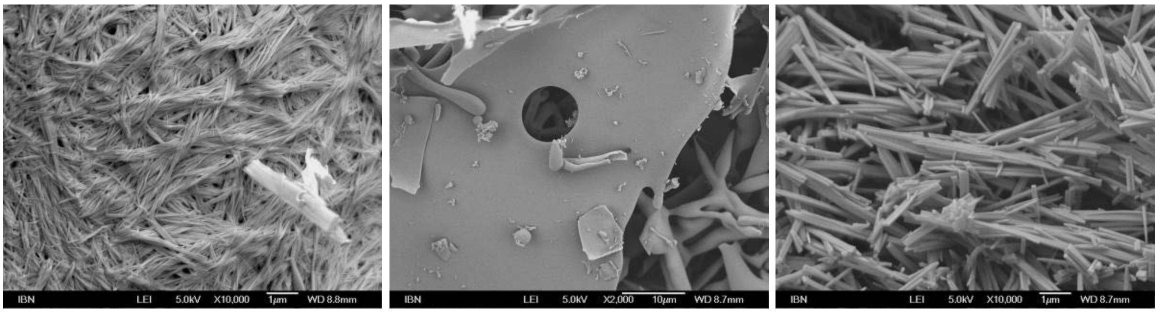

Figure S7-3. Electron micrographs of Ac-YYE: (Left) 5 mg/mL; (middle) 20 mg/mL, supernatant; (right) 20 mg/mL, precipitate

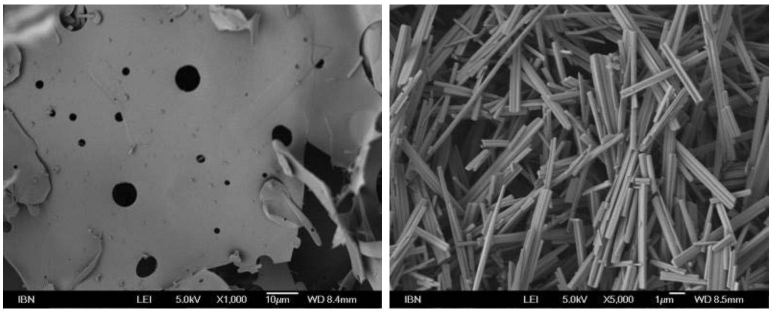

Figure S7-4. Electron micrographs of Ac-YYD-NH_2: (Left) 5 mg/mL, supernatant; (right) 5 mg/mL, precipitate

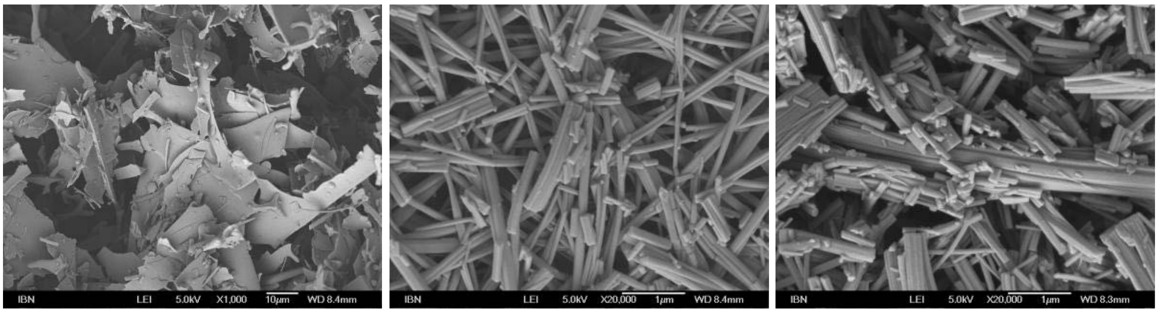

Figure S7-5. Electron micrographs of Ac-YYN: (Left) 5 mg/mL; (middle) 20 mg/mL; (right) 40 mg/mL

Table S1. Details of crystallization, data collection and refinement

**Crystal data**	
Chemical formula	$C_{19}H_{33}N_3O_7$
M_r	415.48
Crystal system, space group	Monoclinic, *C*2
Temperature (K)	100
a, *b*, *c* (Å)	31.362 (3), 5.4514 (5), 13.3403 (13)
α, β, γ (°)	90, 102.504 (5), 90
V (Å^3)	2226.7 (4)
Z	4
Radiation type	Cu Kα radiation, λ = 1.54178 Å
μ (mm^{-1})	0.79
**Data collection**	
Diffractometer	Bruker Kappa APEX-II CCD diffractometer
θ_{min}, θ_{max} (°)	2.9, 54.2
No. of measured, independent and observed [$I > 2\sigma(I)$] reflections	10179, 2662, 1785
R_{int}	0.112
**Refinement**	
$R[F^2 > 2(F^2)]$, $wR(F^2)$, S	0.059, 0.166, 0.94
No. of reflections	2662
No. of parameters	273
No. of restraints	1
$\Delta\rho_{max, min}$ (e Å^{-3})	0.28, -0.34
Absolute structure	Flack x determined using 559 quotients [(I+)-(I-)]/[(I+)+(I-)][1]
Absolute structure parameter	0.0 (4)

Computer programs: *APEX2*[2], *SAINT*[2], *SHELXD*[3], *SHELXL2013*[4], ShelXle[5], C*oot*[6] and *publCIF*[7]

References

1. Parsons, S.; Flack, H. *Acta Crystallogr., A, Found. Crystallogr.* **2004**, *60*, s61.
2. *PROTEUM2*; Bruker AXS Inc.: Madison, WI, USA, 2007.
3. Sheldrick, G. M. *Acta Crystallographica Section D: Biological Crystallography* **2010**, *66*, 479.
4. Sheldrick, G. M.; Schneider, T. R. In *Methods Enzymol.*; Charles W. Carter Jr, R. M. S., Ed.; Academic Press: 1997; Vol. 277, p 319.
5. Huebschle, C. B.; Sheldrick, G. M.; Dittrich, B. *J. Appl. Crystallogr.* **2011**, *44*, 1281.
6. Emsley, P.; Lohkamp, B.; Scott, W. G.; Cowtan, K. *Acta Crystallogr. D Biol. Crystallogr.* **2010**, *66*, 486.
7. Westrip, S. P. *J. Appl. Crystallogr.* **2010**, *43*, 920.
